# Roles of the gut microbiota in human neurodevelopment and adult brain disorders

**DOI:** 10.3389/fnins.2024.1446700

**Published:** 2024-11-26

**Authors:** Rahul Mallick, Sanjay Basak, Ranjit K. Das, Antara Banerjee, Sujay Paul, Surajit Pathak, Asim K. Duttaroy

**Affiliations:** ^1^A.I. Virtanen Institute for Molecular Sciences, University of Eastern Finland, Kuopio, Finland; ^2^Molecular Biology Division, ICMR-National Institute of Nutrition, Indian Council of Medical Research, Hyderabad, India; ^3^Department of Health and Biomedical Sciences, University of Texas Rio Grande Valley, Brownsville, TX, United States; ^4^Faculty of Allied Health Sciences, Chettinad Hospital and Research Institute, Chettinad Academy of Research and Education, Chennai, India; ^5^Tecnologico de Monterrey, School of Engineering and Sciences, Queretaro, Mexico; ^6^Department of Nutrition, Faculty of Medicine, Institute of Basic Medical Sciences, University of Oslo, Oslo, Norway

**Keywords:** gut microbiota, brain development, brain function, dysbiosis, drugs, DHA, antibiotics, maternal diet

## Abstract

Growing evidence demonstrates the connection between gut microbiota, neurodevelopment, and adult brain function. Microbial colonization occurs before the maturation of neural systems and its association with brain development. The early microbiome interactions with the gut-brain axis evolved to stimulate cognitive activities. Gut dysbiosis can lead to impaired brain development, growth, and function. Docosahexaenoic acid (DHA) is critically required for brain structure and function, modulates gut microbiota, and impacts brain activity. This review explores how gut microbiota influences early brain development and adult functions, encompassing the modulation of neurotransmitter activity, neuroinflammation, and blood-brain barrier integrity. In addition, it highlights processes of how the gut microbiome affects fetal neurodevelopment and discusses adult brain disorders.

## Graphical Abstract

Dysbiosis of the gut microbiota is linked to various brain disorders, including Alzheimer’s disease, Parkinson’s disease, multiple sclerosis, and depression. Maintaining gut microbiota homeostasis may help prevent these conditions. 
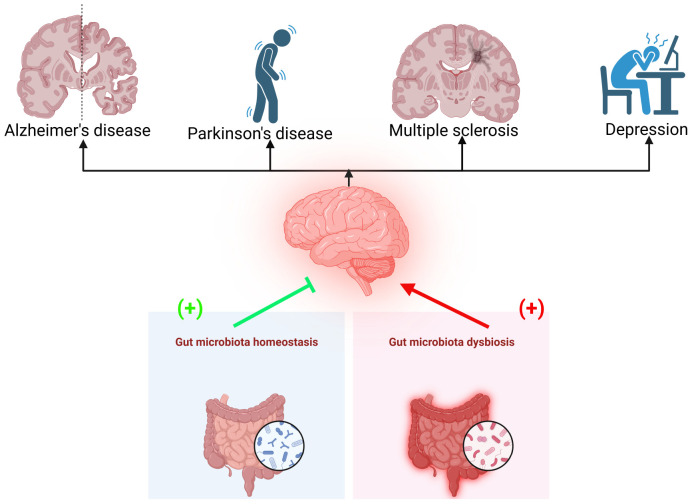

## Introduction

The adult gut microbiota is enriched with *Firmicutes*, *Bacteroidetes*, and *Actinobacteria*. *Bifidobacterium* and *Firmicutes* levels tend to decline in the elderly, with increased levels of *Bacteroidetes* and *Proteobacterium* (Sirisinha, 2016). *Lactobacilli*, *Veillonella*, and *Helicobacter* are the most common bacteria in the gut, while *Bacilli*, *Streptococcaceae*, *Actinomycinaeae*, and *Corynebacteriaceae* reside in the duodenum, jejunum, and ileum. The composition of the intestinal flora in the life course is affected by various factors, including anatomy, gestational age, mode of delivery, breastfeeding, age, antibiotic usage, diet, ethnicity, lifestyle, and environmental exposure (Van Ameringen et al., 2019). The gut microbiome impacts human physiology, including the nervous system (Sirisinha, 2016; Tremlett et al., 2017; Duttaroy, 2021; Adhikary et al., 2024). The gut microbiome is involved in neurogenesis, myelination, microglial maturation, blood-brain barrier (BBB) integrity, and hypothalamic-pituitary-adrenal (HPA)-axis development (Jiang et al., 2017; Basak et al., 2022).

During fetal development, the microbiome’s initial colonization coincides with the nervous system’s growth in a timely, coordinated manner. The gut microbiome and its metabolites regulate early processes of neurodevelopment (Rogers et al., 2016). With aging, a person loses the ability to maintain brain homeostasis because of gut dysbiosis and docosahexaenoic acid, 22:6n-3 (DHA) deficiency (Rogers et al., 2016; Basak et al., 2022). DHA and its metabolites are vital for functional brain development and maintenance (Mallick et al., 2019). The signaling pathways of DHA and its metabolites are involved in neurogenesis, anti-nociceptive effects, anti-apoptotic effects, synaptic plasticity, Ca^2+^ homeostasis in brain diseases, and the functioning of nigrostriatal activities. The evidence of age-associated dysbiosis of gut microbial composition and its contribution to neurocognition disorders is emerging (Dash et al., 2022; Garg and Mohajeri, 2024). Despite these data, unraveling the intricate modulators of the gut-brain axis in developing neurodegenerative diseases, disease onset, and progression could be beneficial in discovering clinically relevant biotherapies to combat the continuous rise in worldwide neurodegenerative diseases. Although several reviews highlighted the gut-brain axis, very few pointed to the impact of exposure to dietary risks and medicine (drug) on gut microbial balance and neuroinflammation in modulating brain development and brain degenerative diseases. This review summarizes recent evidence on how the gut microbiome can influence early human brain development and impaired brain disorders in age-associated dysbiosis.

## Gut microbiota, early brain development and risks of brain dysfunctions

The maternal oral and gut microbiome can influence neurodevelopment during infancy, an essential and dynamic stage of brain growth whose characteristics can predict risk or resilience to neuropsychiatric disorders in childhood or later adulthood (Gomez-Arango et al., 2017). In fact, the connection between maternal gut microbial diversity, brain development, and its function is emerging (Basak et al., 2024). The most direct route of communication between the gut and the brain is the vagus nerve (De la Fuente-Nunez et al., 2018). The CNS interacts with the endocrine, immune, and enteric nervous systems (ENS) to have this intricate regulation. ENS is involved in interconnectivity via the gut-brain axis (GBA). The GBA communicates between the gut microbiome, the gastrointestinal tract, and the nervous system. The interplay between the brain and the gut is crucial as the GBA can modify and regulate cognitive functions and mood, and nutritional compounds transported by the gut that can affect brain development (Sharon et al., 2016; Muhammad et al., 2022). The gut microbiota produces hormones, metabolites, and neurotransmitters, creating a connection between the gut and the brain. Research indicates that diet-associated gut microbial metabolites regulate the relationship between gut microbes and CNS cells (Park and Kim, 2021; Basak et al., 2024). Therefore, dietary strategies centered on signaling molecules associated with gut-brain interaction, including supplementation of SCFAs and tryptophan metabolites, are promising therapeutic options for brain disorders (Gao et al., 2020; O’riordan et al., 2022). A more comprehensive understanding is needed regarding which cells in the brain are affected by gut microbial metabolites to enable the development of more tailored treatments.

Critical temporal control of brain circuit formation, immune cells, and hormone and neurotransmitter signaling pathways regulate neurodevelopment (Kelly et al., 2017; Krol and Feng, 2018). Neurotransmitters stimulate the vagus nerve, microRNA (miRNA), and small non-coding RNA (sncRNA), interacting with the gut microbiota and the CNS. The BBB and intestinal wall permeability regulate communication between the gut microbiota and the CNS.

The germ-free (GF) mice develop abnormal brain functions (Svensson et al., 2015; Tremlett et al., 2017). The colonization of gut microbiota begins before the maturation of neural systems. As a result, it impacts brain function in later life (Bauer et al., 2016). The microbiota interactions along the GBA may modulate brain development. The microbiota influences brain development either prenatally via the mother’s microbiome or postnatally, where factors such as delivery method, breastfeeding, and antibiotic usage. These can alter the healthy gut microbial composition and impact neurodevelopmental processes (Borre et al., 2014; Sharon et al., 2016; Cowan and Cryan, 2021). Perturbation to the mother’s microbiome can affect the development of the fetus via several mechanisms, including metabolic dysregulation (Krol and Feng, 2018; Muhammad et al., 2022). Antibiotic use in pregnant rats resulted in increased anxiety behaviors and reduced sociability in their offspring (Degroote et al., 2016; Tochitani et al., 2016). An association between changes in the vaginal microbiota and, consequently, metabolic processes was reported, a crucial relationship required for proper neurodevelopment (Jasarevic et al., 2015; Muhammad et al., 2022). Moreover, imbalanced microbial colonization leads to metabolic changes and promotes the invasion of opportunist pathogens. After birth, the infant microbiome is enriched with *Lactobacillus* and *Bifidobacterium* contributed by the mother. After weaning, foods change the infant’s gut’s microbial composition. *Firmicutes* are highly abundant in carbohydrate-rich foods, whereas *Bacteroidetes* are primarily present in foods from animal sources. At one year, the gut microbiota has a high quantity of *Akkermansia*, *Veillonella*, *Bacteroides*, and Clostridium. The diversity of the gut microbiota enriches and stabilizes into adulthood (Rinninella et al., 2019).

Table 1 describes the impacts of the microbiome on the neurotransmitters. The gut microbiota can synthesize dopamine, norepinephrine, gamma-aminobutyric acid (GABA), and serotonin, all of which can modulate the CNS (Borre et al., 2014; Socala et al., 2021; Liu et al., 2022). However, it is unclear if these compounds can cross the BBB and directly affect the brain; nevertheless, they can impact the local gut area by reducing proinflammatory cytokines and regulating gut motility, among others (Krol and Feng, 2018). Other metabolites synthesized by the microbiota are the SCFAs produced by the fermentation of dietary fibers (Dalile et al., 2019; Silva et al., 2020; Muhammad et al., 2022). SCFAs modulate the gut by maintaining the integrity of the intestinal barrier, and they can also affect brain development by modifying the BBB permeability via microglia activation and neuroinflammation regulation. Altered gut microbiota with impaired cognitive activity were associated with developmental disorders. For example, impaired rhythmic processing is associated with altered gut microbiota, which was observed in autism spectrum disorder (Lense et al., 2021; Dash et al., 2022).

**TABLE 1:** Microbiome effects on neurotransmitters (Warren et al., 2024).

Microbiome	Effects on neurotransmitters
*Bifidobacterium*	Acetylcholine production ↑ GABA production ↑.
*Enterococcus*	
*Lactobacillus*	
*Streptococcus*	
*Lactobacillus* spp.	Intermediates of GABA/glutamate metabolism ↑
*Bifidobacterium* spp.	
*Bifidobacterium*	Serotonin production ↑.
*Lactobacillus*	
*Streptococcus*	
*Enterococcus*	Serotonin production ↓.

Microbial metabolites such as SCFAs (butyric acid, propionic acid, and acetic acid) determine the neuronal, intestinal, and pancreatic differentiation via embryonic sensing mediated by G protein-coupled receptors, GPR43 and GPR41 (Kimura et al., 2020). The location-based alteration of neurotransmitter systems was reported in the brain of GF mice. Increased 5-hydroxytryptamine (5-HT) levels in the hippocampus were reported in the GF mice (Clarke et al., 2013). Upregulated expression of genes involved with brain plasticity and metabolism, including long-term synaptic potentiation and cyclic adenosine monophosphate (cAMP)-mediated signaling system in these mice, were observed (Diaz Heijtz et al., 2011). The microbiome modulates the serotonergic system in early life. There was a decreased hippocampal expression of the 5-HT_1A_ receptor gene in the dentate gyrus of female GF animals (Neufeld et al., 2011). Expression of the brain-derived neurotrophic factor (BDNF) gene was reduced in the cortex and amygdala in GF mice (Diaz Heijtz et al., 2011). However, the expression of BDNF levels in the hippocampus was inconsistent in GF mice (Clarke et al., 2013). The gut microbiome regulated post-natal neurogenesis in GF mice (Ogbonnaya et al., 2015). However, post-weaning microbiome colonization could not be reversed in these mice. The effect of maternal gut microbiota on embryonic development highlighted its role in shaping the neurometabolic system of the offspring.

The gut microbiome does not only affect brain development *per se* but also alters hippocampus and amygdala function as well. The amygdala is a critical brain region, a key node for gating anxiety, fear-related responses, and social behavior (Ledoux, 2007). GF mice brain shows increased amygdala volume and dendritic hypertrophy in the basolateral amygdala (BLA). The structural and functional alterations of the amygdala are associated with neuropsychiatric and developmental disorders ranging from anxiety (Janak and Tye, 2015) to autism spectrum disorder (Schumann and Amaral, 2006). GF mice endowed with normal microbiota had pyramidal BLA neurons characterized by stubby, thin, and mushroom spines (Janak and Tye, 2015). The amygdala of GF mice had differentially expressed genes, exon utilization, and RNA edits. Early response genes such as FosB (proto-oncogene), Fos, and early growth response 2 (Egr2) were increased in the amygdala with concomitant increased signaling of the transcription factor cAMP response element-binding protein (CREB) in the GF mice (Luczynski et al., 2016b). In GF mice, reduced expression of immune system genes was reported (Luczynski et al., 2016b). These findings indicate the presence of underdeveloped immune system and immature microglia in GF mice (Erny et al., 2015). Since the immune system plays a crucial role in mediating the microbiota’s effects on brain function, the immature immune system in GF may impact brain development.

The gut microbiome also critically regulates pre-frontal cortical myelination. GF mice had hypermyelination and upregulated expression of genes involved in myelination and myelin plasticity events in the pre-frontal cortex (Hoban et al., 2016). The administration of antibiotics during early development in rats did not affect cognitive function, immune or stress-related responses, or anxiety, but visceral hypersensitivity was observed in their later life (O’mahony et al., 2014). The latter was associated with changes in the spinal expression of pain-associated genes. The post-weaning depletion of the gut microbiota by antibiotics showed a relative change in anxiety and cognitive deficits, as observed in GF mice (Desbonnet et al., 2015). Moreover, the depletion reduced anxiety, induced cognitive deficits, changed tryptophan metabolic dynamics, and decreased BDNF, vasopressin, and oxytocin expression in the adult brain. The gut microbiota contributes to obesity as the specific bacteria can extract excessive energy and store it from the ingested nutrients (Turnbaugh et al., 2006; Gohir et al., 2015). In addition, gut microbiota modulates host lipid metabolism (Harris et al., 2012) and immunity (Myles et al., 2013) and thus may promote an aberrant and chronic low-grade inflammation, as observed in obesity .

The maternal gut microbiome promotes fetal thalamocortical axonogenesis by signaling microbe-modulating metabolites to develop neurons in the brain (Vuong et al., 2020). The gut microbiome mediates adverse effects of maternal environments, such as high-fat diet, obesity, dysregulated immune activation, and stress, on the brain development of offspring. Dysbiosis of the maternal gut microbiota, in response to a high-fat diet (Buffington et al., 2016), stress (Jasarevic et al., 2018), and infection (Kim et al., 2017) during pregnancy, is associated with abnormal brain function and behavior in the offspring (Vuong et al., 2017). Manipulating the maternal microbiome and its metabolites during pregnancy produces offspring with altered tactile sensitivity in two aversive somatosensory behavioral tasks, with no overt differences in many other sensorimotor behaviors. The gut microbiota modulates numerous bioactive compounds impacting the intestine, blood, and other organs (Vernocchi et al., 2016). The maternal gut microbiota-regulated fetal brain metabolites can modulate axon outgrowth in thalamic explants of mice and promote fetal thalamocortical axonogenesis in offspring. Microbiome metabolites such as trimethylamine-N-oxide, imidazole propionate, N, N, N-trimethyl-5-aminovalerate, 3-indoxyl sulfate, and hippurate modulate the neurological status and neurite outgrowth (Vuong et al., 2020). However, the molecular mechanisms of these microbial metabolites’ actions are still unknown. A poorly developed maternal microbiome was associated with decreased brain white matter in the offspring (Keunen et al., 2015; Indrio et al., 2017; Lu et al., 2018). Inflammation-induced changes in the maternal gut microbiome disrupted somatosensory cortical architecture in adult mouse offspring (Shin Yim et al., 2017). The maternal gut microbiome modulates host responses to acute insults in the brain, but whether it impacts offspring brain development is unknown.

The microbiomes in malnourished children showed dysregulated expression of axonogenesis proteins, which were alleviated by treatment with microbiota-enriched diets (Gehrig et al., 2019). Epidemiological studies suggested an association between maternal infection and antibiotic use with a greater risk for neurodevelopmental complications in the offspring (Atladottir et al., 2012; Hamad et al., 2019). The interactions between the gut microbiome and fetal nervous system begin prenatally through influences of the maternal gut microbiota on fetal brain metabolomic profiles and gene expression. However, whether early to mid-gestation is a critical period during which the maternal microbiome promotes fetal neurodevelopment is unknown. The entero-mammary axis enables mothers to transfer microbes from the gut to the mammary gland. While breastmilk influences gut microbiota, gut mucosal immunity, and adipose development (Van Den Elsen and Verhasselt, 2021), no fetal brain development data is known.

## Gut microbiota and brain health

**FIGURE 1:**
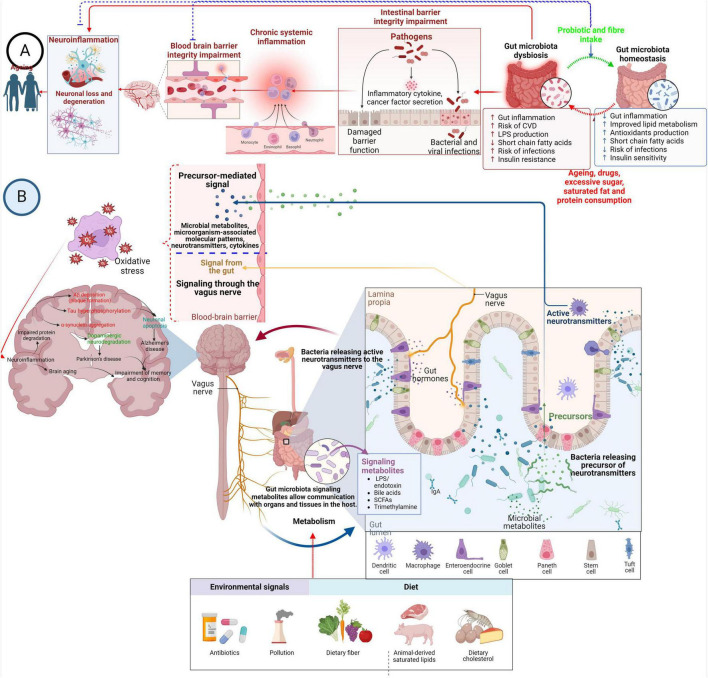
Modulation of neuroinflammation and neurodegenerative diseases by gut microbiota. **(A)** Overconsumption of sugar, saturated fat, protein, medication, and the aging process can disrupt the balance of gut microbes. Still, probiotics and dietary fiber are crucial in maintaining this balance. Dysbiosis in the gut microbiota triggers inflammation in the gut lining, breakdown of tight junctions between cells, and cell death. This leads to migrating pro-inflammatory substances and primed immune cells from the gut into the bloodstream. This process can be reversed by swiftly restoring gut microbiota balance. Prolonged systemic inflammation alters the architecture of the BBB, increasing its permeability and causing collapse. This allows immune cells and pro-inflammatory substances to enter the brain, activating microglia and promoting neuroinflammation and neurodegeneration. **(B)** The microbiota, modulated by environmental signals and diet, plays a pivotal role in shaping the communication between the gut and the brain via its secretions, which include microorganism-associated molecular patterns and microbial metabolites. These molecules contribute to various pathways of gut-brain signaling, including immune modulation, endocrine signaling, neural signaling, and neuroendocrine signaling. Certain substances, such as gamma-aminobutyric acid, act like neurotransmitters, directly impacting the central nervous system through nerve pathways. Additionally, other gut-derived substances like microorganism-associated molecular patterns and short-chain fatty acids can influence the CNS by reducing the permeability of the BBB. Moreover, these microbial molecules can activate resident immune cells or neurons, thereby accelerating the pathophysiology of neurodegenerative diseases through oxidative stress.

The microbiome is an essential functional modulator of the brain and behavior (Vuong et al., 2017). Microbial colonization of the gastrointestinal (GI) tract starts early in birth and matures toward adult composition in three years, closely parallel with brain development. Major depressive disorder is thought to result from the complex interplay of multiple inherited genetic factors and subsequent exposure to a wide range of environmental variables throughout life (Aan Het Rot et al., 2009). There are several different reasons for developing depression; however, studying these ecological variables may be crucial for the prevention and treatment of depressive disorders. The connection between the gut and the brain has, for a long time, been postulated to influence mental health. The connection, the gut-brain axis, is a bidirectional network that links the enteric and central nervous systems (Appleton, 2018). There are now established pathways of the gut-brain axis: neurological, endocrine, humoral/metabolic, and immune. The neurological pathway includes nerves, the enteric nerve system, and neurotransmitters. The endocrine pathway consists of releasing active peptides or stimulating cortisol or norepinephrine, influenced by nutrient availability in the gut. Figure 1 describes the modulation of neuroinflammation and neurodegenerative diseases by gut microbiota.

The immune pathway is related to inflammation and cytokine release from the gut. The metabolic pathway consists of the bacterial metabolites, most notably SCFAs. Therefore, the gut-brain axis and its pathways demonstrate how the intestinal environment can influence brain activity. The gut environment is highly influenced by its microbiota, inducing both beneficial and disadvantageous effects. A balanced microbiota is associated with healthy state and maintenance (Bhattacharjee et al., 2022). Simultaneously with this argument, an unbalanced microbiota can give rise to disease and unhealthy precursors. There are many mechanisms behind probiotics’ beneficial effects, and several suggested theories exist. In the context of the gut-brain axis, one can observe a connection between probiotics and the neurological pathway. Tryptophan is a substrate for different neuroactive metabolites, and one of the main pathways for tryptophan catabolizing is the kynurenine pathway. Some probiotics can indirectly affect the tryptophan availability for serotonin synthesis by reducing the activity of enzymes in the kynurenine pathway located in the gut (Dantzer et al., 2011).

The gut microbiota affects brain function by producing compounds such as cytokines and other inflammatory mediators that target the CNS and ENS (Wood and Galligan, 2004). These ENS maintain intestinal activities. The ENS mediates the enteric neurons and connections to the CNS (Rao and Gershon, 2018). Studies on the gut-brain connection demonstrated a complex communication pathway maintaining the gastrointestinal system. It has various consequences on brain function, including higher cognitive function and motivation (Rhee et al., 2009). The GBA, which is a sophisticated bidirectional communication network between the intestine and the CNS, is where communication occurs between the CNS and intestine (Sudo et al., 2004; Skonieczna Zydecka et al., 2018). Communication routes involve the autonomic nervous system, the neuroendocrine system, the HPA axis, the immune system, and metabolic pathways (Duvallet et al., 2017; Blacher et al., 2019; Burberry et al., 2020). Several neurotransmitters (Yano et al., 2015; O’Keefe, 2016) and metabolites, including SCFAs, secondary bile acids, vitamins, and amino acids (Ellwardt et al., 2016; Engelhardt et al., 2016; Mittal et al., 2017), modulate many immunological pathways (Baj et al., 2019; Dalile et al., 2019), which in turn affect cognition, behavior, and learning, movement (Jenkins et al., 2016; Kennedy et al., 2017; Feng et al., 2021). The GBA regulates the immune system, digestive tract, behavior, stress response, and CNS activity (Savignac et al., 2011; Collins et al., 2012; De Palma et al., 2014; Fond et al., 2015; Pirbaglou et al., 2016; Rincel and Darnaudery, 2020). Notably, advancements in gut microbiota sequencing have revealed a strong relationship between the complex ecosystem and the CNS (Knight et al., 2018). In recent years, there has been increasing interest in studying interactions between the brain and gut microbiota and their bidirectional relationship. In addition, a sedentary lifestyle, obesity, stress, and smoking also lower the beneficial gut microbiota (Benjamin et al., 2012). Dietary composition significantly impacts gut complexity and diversity (Machate et al., 2020). For example, high-fat diets are linked to lower numbers of gram-negative and gram-positive bacteria in the intestine, including *Bifidobacteria*.

## Docosahexaenoic acid, 22:6n-3 and gut microbiota: impacts on brain development and function

Dietary supplementation with DHA may have beneficial effects on behavioral and neurophysiological disorders not only via direct action on the brain structure and function but also due to the alteration of the microbial composition of the gut and an indirect action via the blood-gut axis (Jin et al., 2020). The maternal gut microbiota changes fetal brain metabolites and thus affects the brain development and function of the offspring. The critical role of DHA and the maternal microbiome in offspring neurodevelopment is increasingly appreciated. Higher circulating levels of DHA correlate with optimum microbiota diversity (Jin et al., 2020).

DHA is critically required for the structure and function of the brain (Mallick et al., 2019; Basak et al., 2021; Basak and Duttaroy, 2022). DHA is essential in fetal neurodevelopmental processes, including neuronal differentiation (Katakura et al., 2009), neuritogenesis (Dagai et al., 2009), synaptogenesis (Cao et al., 2009), neurite outgrowth (Calderon and Kim, 2004), and synthesis of neuroprotective metabolites (Kim and Spector, 2018). In utero, n-3 fatty acid deficiency alters fetal brain growth and maturation, reducing neuronal and behavioral plasticity in adulthood (Bhatia et al., 2011; Duttaroy and Basak, 2020; Basak and Duttaroy, 2022). A deficiency of DHA during brain development in the third trimester affects the maturation and plasticity of the brain and its functioning during adult life (Duttaroy, 2004; Mallick et al., 2019).

n-3 PUFA (polyunsaturated fatty acid) deficiency also increases tumor necrosis factor-alpha (TNF-α) and lowers glutamate receptors in the CNS (Kitajka et al., 2004). Moreover, low DHA levels and reduced telencephalon structure were observed in the hippocampus of n-3 PUFA-depleted mice (Coti Bertrand et al., 2006). Brain activities such as learning, motor skills, and monoamine transmission were affected during n-3 PUFA deficiency (Carrie et al., 2000). Maternal DHA deficiency may result in gender-specific offspring’s brain development since the efficiency of endogenous DHA conversion enzymes differs in males from females. Maternal DHA deficiency affects the offspring’s stress response, anxiety (Bondi et al., 2014), hippocampal neurogenesis (Srinivas et al., 2023) and brain reward activities (Auguste et al., 2018). The deficiency of the n-3 PUFAs induces hypomyelination in the developing brain, predisposing the offspring to acquire anxiety-related disorders (Bernardi et al., 2013; Bondi et al., 2014; Auguste et al., 2018).

Consequently, the placental and brain transport of LCPUFAs for fetal brain development during the last trimester is critical (Duttaroy, 2004; Duttaroy and Basak, 2021). The uptake of maternal fatty acids is mediated by intracellular and transmembrane proteins such as fatty acid translocase (FAT/CD36), fatty acid-transport proteins (FATPs), plasma membrane fatty acid-binding proteins (FABPpm), and cytoplasmic fatty acid-binding proteins (FABPs) in the placenta (Campbell et al., 1998; Crabtree et al., 1998; Dutta-Roy, 2000; Johnsen et al., 2009). These proteins are also involved in DHA uptake in the brain (Crabtree et al., 1998; Duttaroy, 2009). MFSD2a (major facilitator superfamily domain-containing protein 2a) a specific protein, can transport plasma lysophosphatidylcholine (LPC)-DHA, but not other DHA forms, across the BBB to the neuron (Basak et al., 2021; Duttaroy and Basak, 2021). Dysregulated placental fatty acid transport carries increased risks of impaired neurodevelopment (Sánchez-Campillo et al., 2020; Basak and Duttaroy, 2022) and cardiometabolic risks (Gómez-Vilarrubla et al., 2021) in the offspring.

DHA-fed rats showed increased BDNF, glutamate ionotropic receptor (GluR2), N-methyl D-aspartate receptor subtype 2B (NR2B), and Tropomyosin receptor kinase B (TrkB) expression in rat brains might promote enhanced memory in rats (Dyall et al., 2007; Bhatia et al., 2011). Collectively, evidence suggests DHA stimulates gene expression directly or by modulating transcription factors of several membrane-associated mediators in brains that may regulate learning and memory functions. In addition, the deficiency of n-3 PUFA resulted in altered dopamine transmission in the brain, probably deranged neurogenesis (Tang et al., 2018). The complex interaction between several brain disorders and intestinal microflora and emotional disorders such as depression, anxiety, and stress in n-3 PUFA deficiency has been demonstrated a relation with gut microbiota alterations (Yang et al., 2021). Bacterial colonization of different species can potentially alter brain functions, and in turn, the central nervous system is speculated to influence the gut microbial composition indirectly (Taylor and Holscher, 2020). Interestingly, the differences in prevalence of some mental disorders between the sexes may also be linked to differences in the microbiota. Studies in mice, for example, show that DHA can lead to reductions in symptoms of anxiety and depression in socially isolated males but not in females, and this is linked to the microbiome (Menni et al., 2017).

## Chronic inflammation, aging and brain dysfunctions

The gut microbiota plays a crucial role in human aging, influencing neuroinflammation immune function, and neurodegenerative diseases (Thevaranjan et al., 2017). Chronic inflammation, marked by proinflammatory biomarkers, is linked to various diseases and aging (Furman et al., 2019), often associated with systemic chronic inflammation (SCI) and inflammaging. SCI is sustained by immune activation associated with chronic diseases, while inflammaging refers to low-grade inflammation in aging (Mou et al., 2022). Both processes involve changes in immune cells and the release of proinflammatory molecules. Recognizing SCI early may help delay aging-related health issues. The BBB and neurovascular unit (NVU) are crucial for brain health and disease (Mark and Miller, 1999). Chronic inflammation compromises BBB integrity, contributing to cerebrovascular disorders. Inflammatory cytokines disrupt endothelial junctions, increasing BBB permeability (Mark and Miller, 1999; Blecharz-Lang et al., 2018). Immune cell migration exacerbates BBB damage, facilitated by cytokines and matrix metalloproteinases. Maintaining BBB integrity involves various factors, including cytokines, vascular endothelial growth factor (VEGF), and reactive oxygen species (ROS). Chronic inflammation, driven by environmental or lifestyle factors, plays also a pivotal role in brain diseases. Strengthening BBB integrity may mitigate neurodegenerative disease progression (Huppert et al., 2010; Blecharz-Lang et al., 2018).

Immune surveillance in the brain parenchyma maintains neuronal homeostasis, with the deep cervical lymph nodes playing a role in priming adaptive immune responses against CNS antigens. The BBB regulates inflammation, and changes in BBB permeability contribute to neuroinflammatory diseases. Chemokines are crucial in BBB integrity and immune cell migration into the CNS. Pro-inflammatory cytokines are elevated in metabolic syndrome and other diseases, impacting BBB integrity and neuroinflammation. Microglia activation is a hallmark of neuroinflammation and can lead to BBB dysfunction. Multiple sclerosis (MS) exemplifies how chronic systemic inflammation affects neuroinflammation via BBB dysregulation. Th17 cells and IL-17 are implicated in multiple sclerosis pathogenesis (Huppert et al., 2010; Surendar et al., 2011; Ohman et al., 2013; Waisman et al., 2015). Understanding immune cell trafficking across the BBB could offer diagnostic insights into neuroinflammatory diseases.

Brain aging, a key indicator of cognitive decline, adds complexity to the pathogenesis of neuroinflammation and neurodegenerative disease (NF&ND) (Cornejo and Von Bernhardi, 2016; Senatorov et al., 2019). Aging and inflammaging negatively affect brain function, with accelerated NF&ND accompanying brain aging. Dysfunctional microglia contribute to chronic neurodegeneration, characterized by hyperactivated transforming growth factor beta (TGFβ) signaling in astrocytes. Microglia activation is a hallmark of NF&ND and aging, with aged microglia showing compromised migration, heightened proinflammatory responses, and altered sensing abilities (Cornejo and Von Bernhardi, 2016). Peripheral T-helper 17 (Th17) lymphocytes play a role in inflammatory disease pathogenesis and can transmigrate across the BBB, contributing to CNS inflammation. BBB dysfunction and energy metabolism disturbances may initiate NF&ND, suggesting potential targets for prevention.

The breakdown of the BBB occurs in aging humans and rodents, starting in middle age and progressing throughout life (Montagne et al., 2019). BBB dysfunction is considered a hallmark of neurological diseases and an early biomarker of cognitive dysfunction. Preclinical studies suggest BBB disruption precedes neurodegeneration in Alzheimer’s disease (AD) (Lin et al., 2021). Changes in BBB permeability, even to small molecules, are associated with mild cognitive impairment. Advanced imaging techniques aid in detecting BBB permeability changes in neurodegenerative diseases. Microglia play a central role in neuroinflammation and BBB integrity (Lin et al., 2021). Dysregulation of microglial function and abnormal brain oxygen supply may contribute to BBB disruption and cognitive decline. BBB disruption is a hallmark of neuroinflammatory and neurodegenerative diseases, but establishing clinical criteria remains challenging.

The glycocalyx on BBB endothelial cells (BECs) prevents macromolecule extravasation and leukocyte adhesion. Chronic inflammation disrupts the glycocalyx, potentially contributing to neuroinflammation and brain aging (Sampei et al., 2021). Lower expression of pattern recognition and chemokine receptors in BECs helps mitigate neuroinflammation. Inflammasome activation in BECs responds to circulating cytokines, potentially influencing neuroinflammation. Inflammasome mechanisms exist in other neurovascular unit members, such as pericytes and astrocytes, suggesting a complex interplay in neuroinflammatory responses (Sampei et al., 2021; Mou et al., 2022). The NVU involves interactions among BECs, pericytes, astrocytes, microglia, and neurons to maintain brain homeostasis. These interactions are crucial for NVU stability, with cytokine crosstalk influencing NVU integrity (Nagyoszi et al., 2010; Haarmann et al., 2019). BECs, frontline defenders against neurotoxicants, interact with pericytes and astrocytes to maintain a low proinflammatory profile. Astrocytes actively contribute to BBB fortification through Sonic Hedgehog (SHH) secretion, regulating BECs and pericytes. Pericytes also play a central role in BBB maintenance and response to inflammation, potentially influencing neuroinflammatory diseases (Mou et al., 2022).

Chronic inflammation disrupts the NVU through various factors, including cytokines, gut microbiota metabolites, and immune cells, impacting BBB integrity. Cytokines like interleukin-1β (IL-1β) and TNF-α influence BBB permeability, while chemokines prime endothelial cells for leukocyte trafficking. Astrocytes and pericytes also respond to inflammation, affecting BBB stability (Nagyoszi et al., 2010). Additionally, aging exacerbates BBB changes, influencing brain microvessel permeability, and astrocyte characteristics (Mou et al., 2022) . Chronic inflammation disrupts the NVU by affecting signaling pathways like Wnt/β-catenin and Sonic Hedgehog (SHH). Inflammatory factors disturb BBB integrity by interfering with Wnt signaling, leading to increased immune cell transmigration. Similarly, IL-1β inhibits astrocyte SHH signaling, promoting BBB disruption and neuroinflammation. The interaction between inflammation and these pathways remains complex and requires further investigation.

## Chronic brain diseases and gut dysbiosis

The human gut microbiota, a complex ecosystem of bacteria that live in the gastrointestinal tract, has received considerable interest for its role in many physiological activities, such as metabolism, immunology, and neurological health. Emerging evidence points to a bidirectional link between the gut microbiota and the CNS, influencing the etiology and progression of chronic brain diseases (Cryan and Dinan, 2015; Sharon et al., 2016). Due to disruption of the gut vascular barrier, inflammation can spread through lymphatic drainage and systemic circulation. Lymphatic fluid carries primed immune cells from the intestine to distant sites, including the brain (Thielke et al., 2003; Shale et al., 2013). The gut vascular unit function resembles the BBB, balancing nutrient absorption and barrier function. Disruption of the gut vascular barrier allows pathogens to enter circulation, contributing to systemic inflammation and the gut-liver-brain axis (Mou et al., 2022). The interaction between gut microbiota and immunity impacts inflammation spread. Dysbiosis can disrupt the intestinal barrier, intensifying inflammation locally and systemically (Chung et al., 2012; Mayassi et al., 2019; Al Bander et al., 2020). Gut virome alterations, dietary habits, and microbial metabolites further influence inflammation. Gut microbiota induces systemic inflammation and releases neurotoxic metabolites, affecting conditions like multiple sclerosis (Mou et al., 2022). Numerous studies have implicated alterations in gut microbiota composition, termed dysbiosis, in the pathophysiology of chronic brain disorders such as Alzheimer’s disease, Parkinson’s disease, multiple sclerosis, and mood disorders (Jiang et al., 2017). Dysbiosis is characterized by microbial diversity, abundance, and metabolic activity changes, leading to systemic inflammation, immune dysregulation, and neurotransmitter imbalances (Rogers et al., 2016). For instance, dysbiosis-induced inflammation and disruption of the gut-brain axis have been linked to neuroinflammation, neurodegeneration, and cognitive decline in Alzheimer’s disease and Parkinson’s disease (Sampson et al., 2016). Upon cell activation triggered by exposure to microbes, danger signals, or stress, the inflammasome complex assembles, producing pro-inflammatory cytokines (such as IL-1β and IL-18) and pyroptosis. Evidence indicates a reciprocal relationship between microbiota and inflammasome activation in the brain (Rutsch et al., 2020).

The gut microbiota and its metabolites play a significant role in neuroinflammatory and neurodegenerative diseases (Zhang et al., 2022). Various studies link gut microbiota alterations to the onset and progression of such diseases (Yacyshyn et al., 1996; Buscarinu et al., 2018). Dysbiosis, gut virome changes, dietary habits, and microbial metabolites influence inflammation and disease progression. Although the exact mechanisms remain unclear, interactions between gut microbiota and immune responses in the gut and brain are central to understanding the diseases pathogenesis. Studies show that disturbances in gut microbiota composition contribute to inflammaging, characterized by chronic low-grade inflammation. Centenarians exhibit a distinct gut microbiota associated with reduced inflammation and cognitive decline, while gut microbiota transplantation from elderly individuals exacerbates inflammation and cognitive dysfunction in animal models (Holzer et al., 2017; Thevaranjan et al., 2017). Gut microbiota-induced brain aging is linked to gut dysfunction and systemic inflammation, highlighting the GBA in aging and neurodegeneration (Kim and Jazwinski, 2018; Garcia-Duran et al., 2021). Aging-related changes in circulating immune cells further underscore the role of gut-immune interactions in brain aging (Mou et al., 2022). The bidirectional communication between the gut microbiota and the CNS occurs via various pathways, including the vagus nerve, immune signaling molecules, microbial metabolites, and the enteric nervous system (Sharon et al., 2016).

Preliminary evidence suggests that vagus nerve stimulation holds promise as an adjunctive therapy for treatment-resistant depression, post-traumatic stress disorder, and inflammatory bowel disease. Treatments targeting the vagus nerve elevate the vagal tone and suppress cytokine production, both crucial mechanisms for resilience. Stimulation of vagal afferent fibers in the gut affects monoaminergic brain systems in the brainstem, which are pivotal in mood and anxiety disorders. Additionally, initial evidence indicates that gut bacteria may positively impact mood and anxiety, partly by modulating vagus nerve activity. Given that vagal tone correlates with the ability to regulate stress responses and can be influenced by breathing, its enhancement through practices like meditation and yoga likely contributes to resilience and alleviates mood and anxiety symptoms (Breit et al., 2018). Metabolites such SCFAs, neurotransmitters (e.g., serotonin, dopamine), and neuroactive compounds (e.g., lipopolysaccharides) those are made by gut microbiota, can modulate neuronal activity, synaptic plasticity, and neuroinflammatory responses, thereby influencing brain function and behavior (Cryan and Dinan, 2012).

The bidirectional relationship between chronic brain disorders and gut microbiota dysbiosis underscores the importance of considering the gut-brain axis in the pathogenesis and management of neurological and psychiatric conditions. Disturbances in the simultaneous coordinated process of neuronal as well as gut-microbiome development due to overdoses of antibiotics in infants can lead to an inflammatory state at this critical phase of brain development (Neuman et al., 2018). Compositional alterations in the gut microbiome can result in systemic inflammation and neuroinflammation (Belkaid and Hand, 2014). Moreover, the microbiome also plays an essential role in microglial maturation. It can modulate glial activation in the CNS, which is also considered a regulating factor of neuroinflammation in the CNS (Abdel-Haq et al., 2019). All these events during the developmental process established a foundation for the onset of several brain disorders. Investigating intricate pathways of the microbiome-gut-brain-immune axis in developing neurodegenerative diseases, disease onset, and progression will be beneficial in discovering clinically relevant targeted biotherapies to combat the continuous rise in worldwide neurodegenerative diseases. Further studies are required to elucidate specific molecular signaling pathways that underlie neuronal development, this knowledge is essential for developing personalized microbiome therapeutics. Future investigations into the intricate interplay between the gut microbiota and the CNS not only promise to advance our understanding of disease mechanisms but also hold the potential to revolutionize patient care through innovative therapeutic strategies.

## Effects of antibiotic or drugs on gut-microbiota-brain axis: impact on brain structure and function

Antibiotics impact on gut microbiota composition and diversity. Depending on their structure, dose, and exposure time, antibiotics reduce the diversity and abundance of the gut microbiome. The use of antibiotics in intrapartum increases the number of *Bacteroides* and *Enterobacteria* while decreasing *Bacteroidetes* in the newborn gut microbiota (Sanchorawala et al., 2017). Antibiotic-induced dysbiosis disturbs gut-brain communication and influences behavior in mice, demonstrating the influence of gut microbiota on brain function (Dahiya and Nigam, 2023; Wan et al., 2023). The antibiotic-induced dysbiosis and GF animals established the GBA’s relation with brain function. Antibiotics deplete the gut microbiota, resulting in decreased neurogenesis in adult animals. The antibiotic effect was reversed by physical activity and or consumption of a probiotic cocktail (Ogbonnaya et al., 2015). The use of antibiotics is negatively associated with the expression of hippocampal BDNF and the recognition memory of mice, as antibiotic use decreased the gut microbial diversity and population in infants (Fröhlich et al., 2016). The cognitive deficit was associated with reduced bacteria-derived metabolites in the colon, altered lipid composition, and changing the expression of neuronal signaling receptors such as N-methyl-D-aspartate (NMDA) 2B, tight junction protein, etc. Table 2 describes the impacts of antibiotics on the gut microbiota. GF mice studies highlighted the critical role of the gut microbiota in early brain development (Sampson and Mazmanian, 2015; Luczynski et al., 2016a).

**TABLE 2:** Antibiotics-induced gut microbiota alteration.

Antibiotics	Classes of antibiotics	Effects on gut microbiota	References
Amoxicillin	Bacterial cell-wall disrupting antibiotics	Beta-lactam and glycopeptide antibiotics demonstrated the ability to cause dysbiosis in the gut	Li et al., 2014; Pauwels et al., 2021
Ceftazidime			
Ceftriaxone			
Cloxacillin			
Nitroimidazole- Quinolone	Multi-class antibiotic therapy	• Beta-lactam and glycopeptide antibiotics demonstrated the ability to cause dysbiosis in the gut • In both the vaginal and urine microbiomes, *Lactobacillus iners* increased in abundance while total diversity decreased • Antibiotics-induced microbiome depletion have anti-inflammatory effects in several animal models, suggesting a role for the gut-microbiota-spleen-brain axis in modulating inflammation	Li et al., 2014; Pauwels et al., 2021; Jakobsson and Forsum, 2007; Chee et al., 2020; Wan et al., 2023
Macrolide			
Metronidazole			
Nitrofuran			
Nitroimidazole			
Nitroimidazole	DNA replication blocking antibiotics	Treatment constantly altered the microbial community compositions in the gut	Horn and Robson, 2001; Sokol et al., 2008; Sokol et al., 2009
Nitrofuran			
Quinolone			
Aminoglycoside	Transcription and protein synthesis inhibiting antibiotics	Alterations in the microbiome could exacerbate disruption within the gut microbiome network; Because of the non-antimicrobial properties of macrolides, modifications may involve changes in mucus secretion, ion transport, and inflammatory responses	Xu and Li, 2019; Roubaud-Baudron et al., 2019; Kanoh and Rubin, 2010
Lincosamide			
Macrolides			
Rifamycin			
Tetracycline			

Drug-induced gut dysbiosis can influence brain activity through the microorganisms present in the gut and their metabolites (Garg and Mohajeri, 2024). Almost 15,000 drugs are used for the treatment of various diseases worldwide. Many drugs may induce dysbiosis in addition to their intended pharmacological effects. However, one significant side effect of antibiotics is a severe alteration of gut microbial compositions. Therefore, in addition to their intended effect, these drugs have other effects via altered gut microbiota. Dysbiosis is observed in many different diseases, suggesting a link between the alteration of the microbiome with the disease and its treatments. Thus, drug-induced gut microbiota alterations could influence brain-related diseases. Table 3 shows the drug-induced dysbiosis and brain-disorder-related dysbiosis. Numerous studies showed a strong relationship between antibiotic-induced dysbiosis and impacting GBA. Many drugs, such as antidepressants, statins, and non-steroidal anti-inflammatory drugs etc., have gut-microbiome-altering effects (Lagadinou et al., 2020; Essmat et al., 2023; Zadori et al., 2023; Garg and Mohajeri, 2024). Therefore, there is a connection between the agents with “newly found” antimicrobial properties and their mechanisms of affecting the microbiota-gut-brain axis. Only a few studies exist combining non-antibiotic drug-induced alterations to the microbiota-gut-brain axis. Taking into account all relevant human data and supporting mechanistic data published in preclinical studies showed that metformin, statins, proton-pump inhibitors (PPIs), and nonsteroidal anti-inflammatory drugs (NSAIDs) may alter microbiota-gut-brain-axis and cause depression, multiple sclerosis, Parkinson’s, and Alzheimer’s as examples of neuronal diseases.

**TABLE 3:** Effects of daily prescribed drugs on gut microbiota contributing to brain disorders.

Name of drugs	Effects on gut microbiota	Brain disorders	References
Metformin	*↓ Alistipes*	Depression ↑	Bryrup et al., 2019; Tong et al., 2018; Garg and Mohajeri, 2024
	*↓ Bacteroides*		
	*↓ Clostridium*		
	↑ *Escherichia/Shigella*	Depression ↓	Bryrup et al., 2019; Garg and Mohajeri, 2024
	↑ *Lactobacillus*		
	*↓ Roseburia*		
	↑ *Akkermansia muciniphila*	Multiple sclerosis ↑	Tong et al., 2018; Bryrup et al., 2019; Mueller et al., 2021; Garg and Mohajeri, 2024
	↑ *Bifidobacterium*		
	↑ *Bilophila wadsworthia*		
	↑ *Blautia*		
	↑ *Ruminococcus torques*		
	*↓ Bacteroides*	Multiple sclerosis ↓	Wu et al., 2017; Tong et al., 2018; Garg and Mohajeri, 2024
	*↓ Lactobacillus*		
	↑ *Akkermansia muciniphila*	Parkinson’s disease ↑	Forslund et al., 2015; Wu et al., 2017; Tong et al., 2018; Bryrup et al., 2019; Garg and Mohajeri, 2024
	*↓ Alistipes*		
	↑ *Bifidobacterium*		
	↑ *Bifidobacterium adolescentis*		
	↑ *Escherichia*		
	↑ *Lactobacillus*		
	*↓ Roseburia*	Parkinson’s disease ↓	Garg and Mohajeri, 2024; Mueller et al., 2021
	↑ *Escherichia/Shigella*	Alzheimer’s disease ↑	Wu et al., 2017; Bryrup et al., 2019; Mueller et al., 2021; Garg and Mohajeri, 2024
	↑ *Bifidobacterium*	Alzheimer’s disease ↓	Wu et al., 2017; Garg and Mohajeri, 2024
Statins	↑ *Bacteroides*	Depression ↑Click or tap here to enter text.	Garg and Mohajeri, 2024; Khan et al., 2018; Vieira- Silva et al., 2020
	*↓ Desulfovibrio*		
	*↓ Faecalibacterium*	Depression ↓Click or tap here to enter text.	Khan et al., 2018; Garg and Mohajeri, 2024
	↑ *Ruminococcus*		
	↑ *Akkermansia/muciniphila*	Multiple sclerosis ↑	Khan et al., 2018; Garg and Mohajeri, 2024
	*↓ Bilophila wadsworthia*		
	↑ *Ruminococcus*		
	↑ *Bacteroides*	Multiple sclerosis ↓	Khan et al., 2018; Garg and Mohajeri, 2024
	*↓ Collinsella*		
	↑ *Streptococcus*		
	*↓ Desulfovibrio*	Parkinson’s disease ↑	Khan et al., 2018; Garg and Mohajeri, 2024
	↑ *Ruminococcaceae*		
	*↓ Streptococcus*		
	↑ *Verrucomicrobiaceae*		
	*↓ Faecalibacterium*	Parkinson’s disease ↓	Khan et al., 2018; Vieira- Silva et al., 2020; Garg and Mohajeri, 2024
	↑ *Faecalibacterium prausnitzii*		
	↑ *Ruminococcaceae*	Alzheimer’s disease ↑	Khan et al., 2018; Garg and Mohajeri, 2024
	*↓ Streptococcus*		
	↑ *Faecalibacterium prausnitzii*	Alzheimer’s disease ↓	Khan et al., 2018; Garg and Mohajeri, 2024
Proton pump inhibitors	*↓Clostridium*	Depression ↑	Garg and Mohajeri, 2024; Naito et al., 2018; Wauters et al., 2021
	↑ *Holdemania*		
	*↓ Selenomonas*		
	↑ *Streptococcaceae*		
	↑ *Streptococcus*		
	*↓ Turicibacter*		
	↑ *Bacteroidaceae*	Depression ↓	Naito et al., 2018; Garg and Mohajeri, 2024
	*↓ Faecalibacterium*		
	↑ *Lactobacillus*		
	↑ *Ruminococcus*		
	*↓ Actinomyces*	Multiple sclerosis ↑	Wauters et al., 2021; Clooney et al., 2016; Naito et al., 2018; Garg and Mohajeri, 2024
	↑ *Blautia*		
	↑ *Dorea*		
	*↓ Haemophilus*		
	*↓ Megasphaera*		
	*↓ Pseudoflavonifractor capillosus*		
	↑ *Ruminococcus*		
	↑ *Streptococcus*		
	*↓ Prevotella*	Multiple sclerosis ↓	Clooney et al., 2016; Naito et al., 2018; Wauters et al., 2021; Garg and Mohajeri, 2024
	↑ *Megasphaera*	Parkinson’s disease ↑	Naito et al., 2018; Garg and Mohajeri, 2024
	*↓ Porphyromonas*		
	↑ *Streptococcus*		
	↑ *Dorea*	Parkinson’s disease ↓	Naito et al., 2018; Garg and Mohajeri, 2024
	*↓ Faecalibacterium*		
	↑ *Veillonella parvula*		
	↑ *Streptococcus*	Alzheimer’s disease ↑	Naito et al., 2018; Garg and Mohajeri, 2024
	↓ Faecalibaeterium	Alzheimer’s disease ↓	Naito et al., 2018; Garg and Mohajeri, 2024
Non-steroidal anti-inflammatory drugs	↑ *Bacteroides*	Depression ↑	Rogers and Aronoff, 2016; Maseda and Ricciotti, 2020; Garg and Mohajeri, 2024
	↑*Barnesiella*		
	↑ *Enterobacteriaceae*		
	↑ *Bacteroides*	Multiple sclerosis ↓	Maseda and Ricciotti, 2020; Garg and Mohajeri, 2024
	↑ *Prevotella*		
	↑ *Enterobacteriaceae*	Parkinson’s disease ↑	Rogers and Aronoff, 2016; Garg and Mohajeri, 2024
	↑ *Ruminococcaceae*		
	↑ *Bacteroides*	Parkinson’s disease ↓	Maseda and Ricciotti, 2020; Garg and Mohajeri, 2024
	↑ *Prevotella*		
	↑ *Bacteroidetes*	Alzheimer’s disease ↑	Rogers and Aronoff, 2016; Maseda and Ricciotti, 2020; Garg and Mohajeri, 2024
	↑ *Prevotellaceae*		
	↑ *Ruminococcaceae*		

*Ruminococcus* spp., an advantageous SCFA producer, is elevated in all the drug users and diseases mentioned above. *Prevotella* and *Akkermansia muciniphila* all have lipopolysaccharides (LPS) in their cell walls and produce beneficial SCFAs. *Escherichia*, also an LPS carrier, can metabolize 5-HT as a precursor for extracellular amyloids (Essmat et al., 2023; Garg and Mohajeri, 2024). Genus *Clostridia* has toxicogenic species such as *Clostridium difficile* and also SCFA-producer species. Depression has depleted levels of SCFAs and GABA. However, *Bacteroides* spp. are increased. They would be in abundance if the disease is correlated with the use of statins and NSAIDs. Bacteroides are GABA and SCFA producer and have LPS in their cell walls. These examples suggest that the relative abundances of the bacterial populations to each other and their interplay would influence the microbiota-gut-brain axis and not specific bacterial strains. In addition, the drugs discussed are frequently taken together in comorbid patients (Zadori et al., 2023). Thus, their effects may counteract each other or intensify the respective changes in bacterial populations and associate positively or negatively with dysbiosis in neurological diseases. Depending upon the study, the bacterial specimens were either from the upper, middle, or lower gastrointestinal tract or stool samples.

In addition, the fecal bacterial samples from humans in antibiotic-treated mice were transplanted to support their clinical studies’ results further. There was a link between using medicines and microbiota-gut-brain-axis and between microbiota-gut-brain-axis and neuronal diseases, respectively (Carabotti et al., 2015). Future studies could focus on the implications of medicinal drugs used to treat somatic diseases and their gut-altering effects on the microbiota-gut-brain axis influencing the onset or progression of brain-related disorders. Since the gut microbiome changes various environmental and lifestyle factors, comorbid elderly people should also be considered in such studies. While taking more than one medication and having a poor diet due to a lack of appetite, one might expect different changes in the gut microbiota of older people. However, the search for the correlation between daily prescribed drugs-induced dysbiosis and their implications on brain-related disorders via the microbiota-gut-brain axis is still a new topic (Loh et al., 2024). Enough data on these correlations does not exist to derive any definitive conclusions. Closing this knowledge gap may result in new critical perspectives for better understanding the bidirectional communication of the microbiota-gut-brain axis and treating patients with the respective individualized treatment with novel therapies.

Consuming pharmaceuticals affects the GBA. Moreover, proton pump inhibitors (PPIs) have been linked to changes in gut microbiota composition. PPI use was associated with changes in gut microbial diversity and increased abundance of potentially dangerous bacteria, indicating a possible mechanism by which PPIs may alter gut-brain communication (Jackson et al., 2016). Nonsteroidal anti-inflammatory medicines (NSAIDs) are also linked to gut microbiome dysbiosis. NSAID use was associated with changes in gut microbial composition and increased intestinal permeability in mice, indicating a possible relationship between NSAIDs, gut barrier function, and brain health (Maseda et al., 2019). These findings highlight the complex link between drug-induced alterations in gut microbiota and their potential effects on the gut-brain axis. Understanding these interactions is critical for creating strategies to reduce medicines’ adverse effects on gut and brain health and improve therapeutic outcomes.

## Probiotics and brain health: human trials

The association between gut microbiota and brain functions and behavior has emerged as an important research area. The gut microbiota and its metabolites may impact the immune and CNS via substances such as SCFAs, serotonin, and GABA (Huang et al., 2016; Kelly et al., 2016; Mu et al., 2016). Correlations between human fecal microbiota and depression were reported (Naseribafrouei et al., 2014). An increased fecal bacterium was observed in the depressed compared with the control group (Jiang et al., 2015). The elevated HPA axis responses and depression were reversed in the rat model by administering *Bifidobacterium infantis* (Desbonnet et al., 2010). The Beck Depression Inventory (BDI) and State-Trait Anxiety Inventor (STAI) trait scores were significantly decreased in the probiotic group (Kazemi et al., 2019). However, the co-supplementation showed a significant decrease in BDI, STAI-trait, and STAI-state scores compared with placebo (Moludi et al., 2022). After adjusting for baseline levels and confounding factors, secondary outcomes showed a substantial reduction in inflammatory markers such as LPS and TNF-α levels in the probiotic group.

Supplementation containing *Lactobacillus acidophilus*, *Lactobacillus casei*, *Bifidobacterium bifidum*, and inulin significantly decreased anxiety, depression, and stress scores (Depression Anxiety Stress Scale-21) (Hadi et al., 2019). In another study compared with the probiotic group received *Lactobacillus acidophilus*, *Bifidobacterium bifidum*, *Bifidobacterium lactis* and *Bifidobacterium longum*, the synbiotic group [received the same strains with fructooligosaccharide (FOS), galactooligosaccharides (GOS), and inulin] had a significant decrease in Hospital Anxiety and Depression Scale (Haghighat et al., 2021). Venkataraman et al. (2021) investigated the effects of *Bacillus coagulants*, *Lactobacillus rhamnosus*, *Bifidobacterium lactis*, *Lactobacillus Plantarum*, *Bifidobacterium breve*, and *Bifidobacterium infantis* among students. There was a significant reduction in BDI and STAI scores in the probiotic group. Secondary outcomes included a decrease in the morning serum cortisol levels. The impact of probiotics on the HPA and mental health involving 75 participants was investigated (Mohammadi et al., 2016). The probiotic group received a daily yogurt containing strains from *Lactobacillus acidophilus* and *Bifidobacterium lactis*. In contrast, the probiotic capsule group received a daily capsule containing strains from *Lactobacillus casei*, *Lactobacillus acidophilus*, *Lactobacillus rhamnosus*, *Lactobacillus bulgaricus*, *Bifidobacterium breve*, *Bifidobacterium longum*, *Streptococcus thermophilus* in addition to a daily conventional yogurt. General health questionnaires (GHQ) and depression anxiety, and stress scores (DASS) significantly improved in both the probiotic yogurt and probiotic capsule groups. There were no significant improvements in kynurenine and kynurenine/tryptophan ratio in the probiotic yogurt or capsule groups. No effect was observed on the HPA, but a substantial improvement in mental health with probiotic supplementation was reported. The probiotic effect of vitamin D for 12 weeks was investigated on mental health in 60 type 2 diabetic patients with coronary heart disease (Raygan et al., 2018). The probiotic strains from *Lactobacillus acidophilus*, *Bifidobacterium bifidum*, *Lactobacillus reuteri*, and *Lactobacillus fermentum*, plus vitamin D, showed a significant improvement in BDI total score, Beck anxiety Inventory (BAI) score and GHQ compared with the placebo group. The serum hs-C-Reactive Protein (CRP) level was reduced in the probiotic group compared with the placebo. There was a significant decrease in the probiotic group’s mean break division inventory score. The study was conducted for 8 weeks in 40 patients with major depressive disorder who received one daily capsule containing *Lactobacillus acidophilus*, *Lactobacillus casei*, and *Bifidobacterium bifidum* (Akkasheh et al., 2016).

## Conclusion

The gut microbiota-brain axis (GBA) communicates between the gut microbiome, the gastrointestinal tract, and the nervous system. The earliest data from germ-free models suggested the critical role of the gut microbiota in deciding early brain development. The influence of maternal gut microbiota on embryonic development suggested its role in shaping the neurometabolic axis and maturing the offspring’s immune system. Gut microbiota, consisting of diverse microbial communities, profoundly affects the CNS through the GBA. Maternal composition of the intestinal flora is affected by multiple factors, including gestational age, mode of delivery, breastfeeding, age, antibiotic usage, ethnicity, lifestyle, environment, and others. The maternal gut microbiome can modulate host responses differentially to acute insults, including malnourishment during in-utero brain development, which can result in a mark for altered brain performance and functions. Such changes in the gut microbiome can influence early human brain development and carry a risk for brain disorders in age-associated dysbiosis. Thus, the maternal microbiome can affect neurodevelopment during infancy, residency for the initial brain growth spurt that can predict risk or resilience to neuropsychiatric disorders later.

Dysbiosis increases susceptibility to brain disorders and neurocognitive function. Understanding the role of gut microbiota dysbiosis in chronic brain disorders holds therapeutic promise for developing novel interventions targeting the gut microbiome. Strategies in restoring microbial homeostasis, such as dietary modifications, prebiotics, probiotics, fecal microbiota transplantation, and microbial-based therapeutics, have shown potential in preclinical and clinical studies for mitigating neuroinflammation, improving cognitive function, and facilitating psychiatric symptoms. However, limited clinical data is available to elucidate the mechanisms underlying gut-brain interactions and optimize the efficacy and safety of microbiota-targeted interventions in diverse ethnicities and populations. Further research is warranted on the causality and mechanisms involved in tracing the gut-brain axis and how this connection can be used in the treatment of mental health disorders such as depression, anxiety, and stress.

## References

[B1] Aan Het Rot M. Mathew S. J. Charney D. S. (2009). Neurobiological mechanisms in major depressive disorder. *CMAJ* 180 305–313. 19188629 10.1503/cmaj.080697 PMC2630359

[B2] Abdel-Haq R. Schlachetzki J. C. M. Glass C. K. Mazmanian S. K. (2019). Microbiome-microglia connections via the gut-brain axis. *J. Exp. Med.* 216 41–59. 10.1084/jem.20180794 30385457 PMC6314531

[B3] Adhikary S. Esmeeta A. Dey A. Banerjee A. Saha B. Gopan P. (2024). Impacts of gut microbiota alteration on age-related chronic liver diseases. *Dig. Liver Dis.* 56 112–122. 10.1016/j.dld.2023.06.017 37407321

[B4] Akkasheh G. Kashani-Poor Z. Tajabadi-Ebrahimi M. Jafari P. Akbari H. Taghizadeh M. (2016). Clinical and metabolic response to probiotic administration in patients with major depressive disorder: A randomized, double-blind, placebo-controlled trial. *Nutrition* 32 315–320. 10.1016/j.nut.2015.09.003 26706022

[B5] Al Bander Z. Nitert M. D. Mousa A. Naderpoor N. (2020). The gut microbiota and inflammation: An overview. *Int. J. Environ. Res. Public Health* 17:7618. 10.3390/ijerph17207618 PMC7589951 33086688

[B6] Appleton J. (2018). The gut-brain axis: Influence of microbiota on mood and mental health. *Integr. Med. (Encinitas)* 17 28–32. 31043907 PMC6469458

[B7] Atladottir H. O. Henriksen T. B. Schendel D. E. Parner E. T. (2012). Autism after infection, febrile episodes, and antibiotic use during pregnancy: An exploratory study. *Pediatrics* 130 e1447–e1454. 10.1542/peds.2012-1107 23147969 PMC4451062

[B8] Auguste S. Sharma S. Fisette A. Fernandes M. F. Daneault C. Des Rosiers C. (2018). Perinatal deficiency in dietary omega-3 fatty acids potentiates sucrose reward and diet-induced obesity in mice. *Int. J. Dev. Neurosci.* 64 8–13. 10.1016/j.ijdevneu.2017.09.003 28919371

[B9] Baj A. Moro E. Bistoletti M. Orlandi V. Crema F. Giaroni C. (2019). Glutamatergic signaling along the microbiota-gut-brain axis. *Int. J. Mol. Sci.* 20:1482. 10.3390/ijms20061482 30934533 PMC6471396

[B10] Basak S. Duttaroy A. K. (2022). Maternal PUFAs, placental epigenetics, and their relevance to fetal growth and brain development. *Reprod. Sci.* 30 408–427. 35676498 10.1007/s43032-022-00989-w

[B11] Basak S. Das R. K. Banerjee A. Paul S. Pathak S. Duttaroy A. K. (2022). Maternal obesity and gut microbiota are associated with fetal brain development. *Nutrients* 14:4515. 10.3390/nu14214515 PMC9654759 36364776

[B12] Basak S. Mallick R. Banerjee A. Pathak S. Duttaroy A. K. (2021). Maternal supply of both arachidonic and docosahexaenoic acids is required for optimal neurodevelopment. *Nutrients* 13:2061. 10.3390/nu13062061 PMC8234848 34208549

[B13] Basak S. Mallick R. Navya Sree B. Duttaroy A. K. (2024). Placental epigenome impacts fetal development: Effects of maternal nutrients and gut microbiota. *Nutrients* 16:1860. 10.3390/nu16121860 38931215 PMC11206482

[B14] Bauer K. C. Huus K. E. Finlay B. B. (2016). Microbes and the mind: Emerging hallmarks of the gut microbiota-brain axis. *Cell Microbiol.* 18 632–644. 10.1111/cmi.12585 26918908

[B15] Belkaid Y. Hand T. W. (2014). Role of the microbiota in immunity and inflammation. *Cell* 157 121–141. 24679531 10.1016/j.cell.2014.03.011 PMC4056765

[B16] Benjamin J. L. Hedin C. R. Koutsoumpas A. Ng S. C. Mccarthy N. E. Prescott N. J. (2012). Smokers with active Crohn’s disease have a clinically relevant dysbiosis of the gastrointestinal microbiota. *Inflamm. Bowel Dis.* 18 1092–1100. 10.1002/ibd.21864 22102318

[B17] Bernardi J. R. Ferreira C. F. Senter G. Krolow R. De Aguiar B. W. Portella A. K. (2013). Early life stress interacts with the diet deficiency of omega-3 fatty acids during the life course increasing the metabolic vulnerability in adult rats. *PLoS One* 8 e62031–e62042. 10.1371/journal.pone.0062031 23614006 PMC3629088

[B18] Bhatia H. S. Agrawal R. Sharma S. Huo Y. X. Ying Z. Gomez-Pinilla F. (2011). Omega-3 fatty acid deficiency during brain maturation reduces neuronal and behavioral plasticity in adulthood. *PLoS One* 6:e28451. 10.1371/journal.pone.0028451 22163304 PMC3233581

[B19] Bhattacharjee G. Khambhati K. Gohil N. Singh P. Gohil J. Gautam H. (2022). Gut microbiota in gastrointestinal diseases. *Prog. Mol. Biol. Transl. Sci.* 191 141–151. 36270675 10.1016/bs.pmbts.2022.06.028

[B20] Blacher E. Bashiardes S. Shapiro H. Rothschild D. Mor U. Dori-Bachash M. (2019). Potential roles of gut microbiome and metabolites in modulating ALS in mice. *Nature* 572 474–480. 10.1038/s41586-019-1443-5 31330533

[B21] Blecharz-Lang K. G. Wagner J. Fries A. Nieminen-Kelha M. Rosner J. Schneider U. C. (2018). Interleukin 6-mediated endothelial barrier disturbances can be attenuated by blockade of the IL6 receptor expressed in brain microvascular endothelial cells. *Transl. Stroke Res.* 9 631–642. 10.1007/s12975-018-0614-2 29429002

[B22] Bondi C. O. Taha A. Y. Tock J. L. Totah N. K. Cheon Y. Torres G. E. (2014). Adolescent behavior and dopamine availability are uniquely sensitive to dietary omega-3 fatty acid deficiency. *Biol. Psychiatry* 75 38–46. 10.1016/j.biopsych.2013.06.007 23890734 PMC3858419

[B23] Borre Y. E. O’keeffe G. W. Clarke G. Stanton C. Dinan T. G. Cryan J. F. (2014). Microbiota and neurodevelopmental windows: Implications for brain disorders. *Trends Mol. Med.* 20 509–518. 24956966 10.1016/j.molmed.2014.05.002

[B24] Breit S. Kupferberg A. Rogler G. Hasler G. (2018). Vagus nerve as modulator of the brain-gut axis in psychiatric and inflammatory disorders. *Front. Psychiatry* 9:44. 10.3389/fpsyt.2018.00044 PMC5859128 29593576

[B25] Bryrup T. Thomsen C. W. Kern T. Allin K. H. Brandslund I. Jorgensen N. R. (2019). Metformin-induced changes of the gut microbiota in healthy young men: Results of a non-blinded, one-armed intervention study. *Diabetologia* 62 1024–1035. 10.1007/s00125-019-4848-7 30904939 PMC6509092

[B26] Buffington S. A. Di Prisco G. V. Auchtung T. A. Ajami N. J. Petrosino J. F. Costa-Mattioli M. (2016). Microbial reconstitution reverses maternal diet-induced social and synaptic deficits in offspring. *Cell* 165 1762–1775. 10.1016/j.cell.2016.06.001 27315483 PMC5102250

[B27] Burberry A. Wells M. F. Limone F. Couto A. Smith K. S. Keaney J. (2020). C9orf72 suppresses systemic and neural inflammation induced by gut bacteria. *Nature* 582 89–94. 10.1038/s41586-020-2288-7 32483373 PMC7416879

[B28] Buscarinu M. C. Romano S. Mechelli R. Pizzolato Umeton R. Ferraldeschi M. Fornasiero A. (2018). Intestinal permeability in relapsing-remitting multiple sclerosis. *Neurotherapeutics* 15 68–74. 29119385 10.1007/s13311-017-0582-3 PMC5794695

[B29] Calderon F. Kim H. Y. (2004). Docosahexaenoic acid promotes neurite growth in hippocampal neurons. *J. Neurochem.* 90 979–988. 15287904 10.1111/j.1471-4159.2004.02520.x

[B30] Campbell F. M. Gordon M. J. Dutta-Roy A. K. (1998). Placental membrane fatty acid-binding protein preferentially binds arachidonic and docosahexaenoic acids. *Life Sci.* 63 235–240. 9698032 10.1016/s0024-3205(98)00267-7

[B31] Cao D. Kevala K. Kim J. Moon H. S. Jun S. B. Lovinger D. (2009). Docosahexaenoic acid promotes hippocampal neuronal development and synaptic function. *J. Neurochem.* 111 510–521. 19682204 10.1111/j.1471-4159.2009.06335.x PMC2773444

[B32] Carabotti M. Scirocco A. Maselli M. A. Severi C. (2015). The gut-brain axis: Interactions between enteric microbiota, central and enteric nervous systems. *Ann. Gastroenterol.* 28 203–209. 25830558 PMC4367209

[B33] Carrie I. Clement M. De Javel D. Frances H. Bourre J. M. (2000). Specific phospholipid fatty acid composition of brain regions in mice. Effects of n-3 polyunsaturated fatty acid deficiency and phospholipid supplementation. *J. Lipid Res.* 41 465–472. 10706594

[B34] Chee W. J. Y. Chew S. Y. Than L. T. L. (2020). Vaginal microbiota and the potential of Lactobacillus derivatives in maintaining vaginal health. *Microb. Cell Fact.* 19:203. 10.1186/s12934-020-01464-4 PMC7648308 33160356

[B35] Chung H. Pamp S. J. Hill J. A. Surana N. K. Edelman S. M. Troy E. B. (2012). Gut immune maturation depends on colonization with a host-specific microbiota. *Cell* 149 1578–1593. 10.1016/j.cell.2012.04.037 22726443 PMC3442780

[B36] Clarke G. Grenham S. Scully P. Fitzgerald P. Moloney R. D. Shanahan F. (2013). The microbiome-gut-brain axis during early life regulates the hippocampal serotonergic system in a sex-dependent manner. *Mol. Psychiatry* 18 666–673. 10.1038/mp.2012.77 22688187

[B37] Clooney A. G. Bernstein C. N. Leslie W. D. Vagianos K. Sargent M. Laserna-Mendieta E. J. (2016). A comparison of the gut microbiome between long-term users and non-users of proton pump inhibitors. *Aliment. Pharmacol. Ther.* 43 974–984. 10.1111/apt.13568 26923470

[B38] Collins S. M. Surette M. Bercik P. (2012). The interplay between the intestinal microbiota and the brain. *Nat. Rev. Microbiol.* 10 735–742. 10.1038/nrmicro2876 23000955

[B39] Cornejo F. Von Bernhardi R. (2016). Age-dependent changes in the activation and regulation of microglia. *Adv. Exp. Med. Biol.* 949 205–226. 27714691 10.1007/978-3-319-40764-7_10

[B40] Coti Bertrand P. O’kusky J. R. Innis S. M. (2006). Maternal dietary (n-3) fatty acid deficiency alters neurogenesis in the embryonic rat brain. *J. Nutr.* 136 1570–1575. 10.1093/jn/136.6.1570 16702323

[B41] Cowan C. S. M. Cryan J. F. (2021). The microbiome-gut-brain axis in neurocognitive development and decline. *Mod. Trends Psychiatry* 32 12–25. 34032642 10.1159/000510414

[B42] Crabtree J. T. Gordon M. J. Campbell F. M. Dutta-Roy A. K. (1998). Differential distribution and metabolism of arachidonic acid and docosahexaenoic acid by human placental choriocarcinoma (BeWo) cells. *Mol. Cell. Biochem.* 185 191–198. 10.1023/a:1006852230337 9746226

[B43] Cryan J. F. Dinan T. G. (2012). Mind-altering microorganisms: The impact of the gut microbiota on brain and behaviour. *Nat. Rev. Neurosci.* 13 701–712. 10.1038/nrn3346 22968153

[B44] Cryan J. F. Dinan T. G. (2015). Gut microbiota: Microbiota and neuroimmune signalling-Metchnikoff to microglia. *Nat. Rev. Gastroenterol. Hepatol.* 12 494–496. 10.1038/nrgastro.2015.127 26215386

[B45] Dagai L. Peri-Naor R. Birk R. Z. (2009). Docosahexaenoic acid significantly stimulates immediate early response genes and neurite outgrowth. *Neurochem. Res.* 34 867–875. 10.1007/s11064-008-9845-z 18781386

[B46] Dahiya D. Nigam P. S. (2023). Antibiotic-therapy-induced gut dysbiosis affecting gut microbiota-brain axis and cognition: Restoration by intake of probiotics and synbiotics. *Int. J. Mol. Sci.* 24:3074. 10.3390/ijms24043074 36834485 PMC9959899

[B47] Dalile B. Van Oudenhove L. Vervliet B. Verbeke K. (2019). The role of short-chain fatty acids in microbiota-gut-brain communication. *Nat. Rev. Gastroenterol. Hepatol.* 16 461–478. 31123355 10.1038/s41575-019-0157-3

[B48] Dantzer R. O’connor J. C. Lawson M. A. Kelley K. W. (2011). Inflammation-associated depression: From serotonin to kynurenine. *Psychoneuroendocrinology* 36 426–436. 21041030 10.1016/j.psyneuen.2010.09.012 PMC3053088

[B49] Dash S. Syed Y. A. Khan M. R. (2022). Understanding the role of the gut microbiome in brain development and its association with neurodevelopmental psychiatric disorders. *Front. Cell Dev. Biol.* 10:880544. 10.3389/fcell.2022.880544 PMC9048050 35493075

[B50] De la Fuente-Nunez C. Meneguetti B. T. Franco O. L. Lu T. K. (2018). Neuromicrobiology: How microbes influence the brain. *ACS Chem. Neurosci.* 9 141–150. 10.1021/acschemneuro.7b00373 29220570

[B51] De Palma G. Collins S. M. Bercik P. Verdu E. F. (2014). The microbiota-gut-brain axis in gastrointestinal disorders: Stressed bugs, stressed brain or both? *J. Physiol. (London)* 592 2989–2997. 10.1113/jphysiol.2014.273995 24756641 PMC4214655

[B52] Degroote S. Hunting D. J. Baccarelli A. A. Takser L. (2016). Maternal gut and fetal brain connection: Increased anxiety and reduced social interactions in Wistar rat offspring following peri-conceptional antibiotic exposure. *Prog. Neuropsychopharmacol. Biol. Psychiatry* 71 76–82. 10.1016/j.pnpbp.2016.06.010 27346743 PMC6584952

[B53] Desbonnet L. Clarke G. Traplin A. O’sullivan O. Crispie F. Moloney R. D. (2015). Gut microbiota depletion from early adolescence in mice: Implications for brain and behaviour. *Brain Behav. Immun.* 48 165–173. 25866195 10.1016/j.bbi.2015.04.004

[B54] Desbonnet L. Garrett L. Clarke G. Kiely B. Cryan J. F. Dinan T. G. (2010). Effects of the probiotic Bifidobacterium infantis in the maternal separation model of depression. *Neuroscience* 170 1179–1188. 10.1016/j.neuroscience.2010.08.005 20696216

[B55] Diaz Heijtz R. Wang S. Anuar F. Qian Y. Bjorkholm B. Samuelsson A. (2011). Normal gut microbiota modulates brain development and behavior. *Proc. Natl. Acad. Sci. U.S.A.* 108 3047–3052. 21282636 10.1073/pnas.1010529108 PMC3041077

[B56] Dutta-Roy A. K. (2000). Transport mechanisms for long-chain polyunsaturated fatty acids in the human placenta. *Am. J. Clin. Nutr.* 71 315S–322S. 10617989 10.1093/ajcn/71.1.315s

[B57] Duttaroy A. K. (2004). Fetal growth and development: Roles of fatty acid transport proteins and nuclear transcription factors in human placenta. *Indian J. Exp. Biol.* 42 747–757. 15573522

[B58] Duttaroy A. K. (2009). Transport of fatty acids across the human placenta: A review. *Prog. Lipid Res.* 48 52–61. 19041341 10.1016/j.plipres.2008.11.001

[B59] Duttaroy A. K. (2021). Role of gut microbiota and their metabolites on atherosclerosis, hypertension and human blood platelet function: A review. *Nutrients* 13:144. 10.3390/nu13010144 PMC7824497 33401598

[B60] Duttaroy A. K. Basak S. (2020). Maternal dietary fatty acids and their roles in human placental development. *Prostaglandins Leukot. Essent. Fatty Acids* 155 102080–102088. 32120190 10.1016/j.plefa.2020.102080

[B61] Duttaroy A. K. Basak S. (2021). Maternal fatty acid metabolism in pregnancy and its consequences in the feto-placental development. *Front. Physiol.* 12:787848. 10.3389/fphys.2021.787848 35126178 PMC8811195

[B62] Duvallet C. Gibbons S. M. Gurry T. Irizarry R. A. Alm E. J. (2017). Meta-analysis of gut microbiome studies identifies disease-specific and shared responses. *Nat. Commun.* 8 1–10. 10.1038/s41467-017-01973-8 29209090 PMC5716994

[B63] Dyall S. C. Michael G. J. Whelpton R. Scott A. G. Michael-Titus A. T. (2007). Dietary enrichment with omega-3 polyunsaturated fatty acids reverses age-related decreases in the GluR2 and NR2B glutamate receptor subunits in rat forebrain. *Neurobiol. Aging* 28 424–439. 10.1016/j.neurobiolaging.2006.01.002 16500747

[B64] Ellwardt E. Walsh J. T. Kipnis J. Zipp F. (2016). Understanding the role of T cells in CNS homeostasis. *Trends Immunol.* 37 154–165. 10.1016/j.it.2015.12.008 26775912

[B65] Engelhardt B. Carare R. O. Bechmann I. Laman J. D. Weller R. O. (2016). Vascular, glial, and lymphatic immune gateways of the central nervous system. *Acta Neuropathol.* 132 317–338. 10.1007/s00401-016-1606-5 27522506 PMC4992028

[B66] Erny D. Hrabe De Angelis A. L. Jaitin D. Wieghofer P. Staszewski O. David E. (2015). Host microbiota constantly control maturation and function of microglia in the CNS. *Nat. Neurosci.* 18 965–977. 10.1038/nn.4030 26030851 PMC5528863

[B67] Essmat N. Karadi D. A. Zador F. Kiraly K. Furst S. Al-Khrasani M. (2023). Insights into the current and possible future use of opioid antagonists in relation to opioid-induced constipation and dysbiosis. *Molecules* 28:7766. 10.3390/molecules28237766 38067494 PMC10708112

[B68] Feng Y. Zhou Z. Zheng C. Feng F. Xie F. Wu Z. (2021). Interleukin 17-producing′ T-cell induced demyelination of the brain in angiostrongylus cantonensis infection. *Mol. Neurobiol.* 58 3968–3982. 10.1007/s12035-021-02366-1 33904019

[B69] Fond G. Boukouaci W. Chevalier G. Regnault A. Eberl G. Hamdani N. (2015). The “psychomicrobiotic”: Targeting microbiota in major psychiatric disorders: A systematic review. *Pathol. Biol.* 63 35–42. 10.1016/j.patbio.2014.10.003 25468489

[B70] Forslund K. Hildebrand F. Nielsen T. Falony G. Le Chatelier E. Sunagawa S. (2015). Disentangling type 2 diabetes and metformin treatment signatures in the human gut microbiota. *Nature* 528 262–266. 26633628 10.1038/nature15766 PMC4681099

[B71] Fröhlich E. E. Farzi A. Mayerhofer R. Reichmann F. Jačan A. Wagner B. (2016). Cognitive impairment by antibiotic-induced gut dysbiosis: Analysis of gut microbiota-brain communication. *Brain Behav. Immun.* 56 140–155. 10.1016/j.bbi.2016.02.020 26923630 PMC5014122

[B72] Furman D. Campisi J. Verdin E. Carrera-Bastos P. Targ S. Franceschi C. (2019). Chronic inflammation in the etiology of disease across the life span. *Nat. Med.* 25 1822–1832. 31806905 10.1038/s41591-019-0675-0 PMC7147972

[B73] Gao K. Mu C. L. Farzi A. Zhu W. Y. (2020). Tryptophan metabolism: A link between the gut microbiota and brain. *Adv. Nutr.* 11 709–723. 31825083 10.1093/advances/nmz127 PMC7231603

[B74] Garcia-Duran C. Martinez-Lopez R. Zapico I. Perez E. Romeu E. Arroyo J. (2021). Distinct human gut microbial taxonomic signatures uncovered with different sample processing and microbial cell disruption methods for metaproteomic analysis. *Front. Microbiol.* 12:618566. 10.3389/fmicb.2021.618566 34290676 PMC8287257

[B75] Garg K. Mohajeri M. H. (2024). Potential effects of the most prescribed drugs on the microbiota-gut-brain-axis: A review. *Brain Res. Bull.* 207:110883. 10.1016/j.brainresbull.2024.110883 38244807

[B76] Gehrig J. L. Venkatesh S. Chang H. W. Hibberd M. C. Kung V. L. Cheng J. (2019). Effects of microbiota-directed foods in gnotobiotic animals and undernourished children. *Science* 365:eaau4732. 10.1126/science.aau4732 PMC6683325 31296738

[B77] Gohir W. Ratcliffe E. M. Sloboda D. M. (2015). Of the bugs that shape us: Maternal obesity, the gut microbiome, and long-term disease risk. *Pediatr. Res.* 77 196–204. 10.1038/pr.2014.169 25314580

[B78] Gomez-Arango L. F. Barrett H. L. Mcintyre H. D. Callaway L. K. Morrison M. Nitert M. D. (2017). Contributions of the maternal oral and gut microbiome to placental microbial colonization in overweight and obese pregnant women. *Sci. Rep.* 7:2860. 10.1038/s41598-017-03066-4 28588199 PMC5460277

[B79] Gómez-Vilarrubla A. Mas-Parés B. Díaz M. Xargay-Torrent S. Carreras-Badosa G. Jové M. (2021). Fatty acids in the placenta of appropiate- versus small-for-gestational-age infants at term birth. *Placenta* 109 4–10. 10.1016/j.placenta.2021.04.009 33895685

[B80] Haarmann A. Schuhmann M. K. Silwedel C. Monoranu C. M. Stoll G. Buttmann M. (2019). Human brain endothelial CXCR2 is inflammation-inducible and mediates CXCL5- and CXCL8-triggered paraendothelial barrier breakdown. *Int. J. Mol. Sci.* 20:602. 10.3390/ijms20030602 30704100 PMC6387364

[B81] Hadi A. Sepandi M. Marx W. Moradi S. Parastouei K. (2019). Clinical and psychological responses to synbiotic supplementation in obese or overweight adults: A randomized clinical trial. *Complement. Ther. Med.* 47:102216. 10.1016/j.ctim.2019.102216 31780038

[B82] Haghighat N. Rajabi S. Mohammadshahi M. (2021). Effect of synbiotic and probiotic supplementation on serum brain-derived neurotrophic factor level, depression and anxiety symptoms in hemodialysis patients: A randomized, double-blinded, clinical trial. *Nutr. Neurosci.* 24 490–499. 10.1080/1028415X.2019.1646975 31379269

[B83] Hamad A. F. Alessi-Severini S. Mahmud S. M. Brownell M. Kuo I. F. (2019). Prenatal antibiotics exposure and the risk of autism spectrum disorders: A population-based cohort study. *PLoS One* 14:e0221921. 10.1371/journal.pone.0221921 PMC6715235 31465485

[B84] Harris K. Kassis A. Major G. Chou C. J. (2012). Is the gut microbiota a new factor contributing to obesity and its metabolic disorders? *J. Obes.* 2012:879151. 10.1155/2012/879151 PMC3270440 22315672

[B85] Hoban A. E. Stilling R. M. Ryan F. J. Shanahan F. Dinan T. G. Claesson M. J. (2016). Regulation of prefrontal cortex myelination by the microbiota. *Transl. Psychiatry* 6:e774. 10.1038/tp.2016.42 PMC4872400 27045844

[B86] Holzer P. Farzi A. Hassan A. M. Zenz G. Jacan A. Reichmann F. (2017). Visceral inflammation and immune activation stress the brain. *Front. Immunol.* 8:1613. 10.3389/fimmu.2017.01613 PMC5702648 29213271

[B87] Horn R. Robson H. G. (2001). Susceptibility of the *Bacteroides* fragilis group to newer quinolones and other standard anti-anaerobic agents. *J. Antimicrob. Chemother.* 48 127–130. 10.1093/jac/48.1.127 11418523

[B88] Huang R. Wang K. Hu J. (2016). Effect of probiotics on depression: A systematic review and meta-analysis of randomized controlled trials. *Nutrients* 8:483. 10.3390/nu8080483 PMC4997396 27509521

[B89] Huppert J. Closhen D. Croxford A. White R. Kulig P. Pietrowski E. (2010). Cellular mechanisms of IL-17-induced blood-brain barrier disruption. *FASEB J.* 24 1023–1034. 19940258 10.1096/fj.09-141978

[B90] Indrio F. Martini S. Francavilla R. Corvaglia L. Cristofori F. Mastrolia S. A. (2017). Epigenetic matters: The link between early nutrition, microbiome, and long-term health development. *Front. Pediatr.* 5:178. 10.3389/fped.2017.00178 28879172 PMC5572264

[B91] Jackson M. A. Goodrich J. K. Maxan M. E. Freedberg D. E. Abrams J. A. Poole A. C. (2016). Proton pump inhibitors alter the composition of the gut microbiota. *Gut* 65 749–756. 26719299 10.1136/gutjnl-2015-310861 PMC4853574

[B92] Jakobsson T. Forsum U. (2007). Lactobacillus iners: A marker of changes in the vaginal flora? *J. Clin. Microbiol.* 45:3145. 10.1128/JCM.00558-07 17652481 PMC2045263

[B93] Janak P. H. Tye K. M. (2015). From circuits to behaviour in the amygdala. *Nature* 517 284–292. 25592533 10.1038/nature14188 PMC4565157

[B94] Jasarevic E. Howard C. D. Morrison K. Misic A. Weinkopff T. Scott P. (2018). The maternal vaginal microbiome partially mediates the effects of prenatal stress on offspring gut and hypothalamus. *Nat. Neurosci.* 21 1061–1071. 10.1038/s41593-018-0182-5 29988069

[B95] Jasarevic E. Howerton C. L. Howard C. D. Bale T. L. (2015). Alterations in the vaginal microbiome by maternal stress are associated with metabolic reprogramming of the offspring gut and brain. *Endocrinology* 156 3265–3276. 10.1210/en.2015-1177 26079804 PMC4541625

[B96] Jenkins T. A. Nguyen J. C. D. Polglaze K. E. Bertrand P. P. (2016). Influence of tryptophan and serotonin on mood and cognition with a possible role of the gut-brain axis. *Nutrients* 8:56. 10.3390/nu8010056 26805875 PMC4728667

[B97] Jiang C. Li G. Huang P. Liu Z. Zhao B. (2017). The gut microbiota and Alzheimer’s disease. *J. Alzheimers Dis.* 58 1–15. 28372330 10.3233/JAD-161141

[B98] Jiang H. Ling Z. Zhang Y. Mao H. Ma Z. Yin Y. (2015). Altered fecal microbiota composition in patients with major depressive disorder. *Brain Behav. Immun.* 48 186–194. 25882912 10.1016/j.bbi.2015.03.016

[B99] Jin J. Jin Q. Wang X. Akoh C. C. (2020). High Sn-2 docosahexaenoic acid lipids for brain benefits, and their enzymatic syntheses: A review. *Engineering* 6 424–431.

[B100] Johnsen G. M. Weedon-Fekjaer M. S. Tobin K. R. Staff A. C. Duttaroy A. K. (2009). Long-chain polyunsaturated fatty acids stimulate cellular fatty acid uptake in human placental choriocarcinoma (BeWo) cells. *Placenta* 30 1037–1044. 19880178 10.1016/j.placenta.2009.10.004

[B101] Kanoh S. Rubin B. K. (2010). Mechanisms of action and clinical application of macrolides as immunomodulatory medications. *Clin. Microbiol. Rev.* 23 590–615. 20610825 10.1128/CMR.00078-09 PMC2901655

[B102] Katakura M. Hashimoto M. Shahdat H. M. Gamoh S. Okui T. Matsuzaki K. (2009). Docosahexaenoic acid promotes neuronal differentiation by regulating basic helix–loop–helix transcription factors and cell cycle in neural stem cells. *Neuroscience* 160 651–660. 10.1016/j.neuroscience.2009.02.057 19272428

[B103] Kazemi A. Noorbala A. A. Azam K. Eskandari M. H. Djafarian K. (2019). Effect of probiotic and prebiotic vs placebo on psychological outcomes in patients with major depressive disorder: A randomized clinical trial. *Clin. Nutr.* 38 522–528. 10.1016/j.clnu.2018.04.010 29731182

[B104] Kelly J. R. Clarke G. Cryan J. F. Dinan T. G. (2016). Brain-gut-microbiota axis: Challenges for translation in psychiatry. *Ann. Epidemiol.* 26 366–372. 10.1016/j.annepidem.2016.02.008 27005587

[B105] Kelly J. R. Minuto C. Cryan J. F. Clarke G. Dinan T. G. (2017). Cross talk: The microbiota and neurodevelopmental disorders. *Front. Neurosci.* 11:490. 10.3389/fnins.2017.00490 PMC5605633 28966571

[B106] Kennedy P. J. Cryan J. F. Dinan T. G. Clarke G. (2017). Kynurenine pathway metabolism and the microbiota-gut-brain axis. *Neuropharmacology* 112 399–412. 10.1016/j.neuropharm.2016.07.002 27392632

[B107] Keunen K. Van Elburg R. M. Van Bel F. Benders M. J. (2015). Impact of nutrition on brain development and its neuroprotective implications following preterm birth. *Pediatr. Res.* 77 148–155. 10.1038/pr.2014.171 25314585 PMC4291511

[B108] Khan T. J. Ahmed Y. M. Zamzami M. A. Siddiqui A. M. Khan I. Mehanna M. G. (2018). Atorvastatin treatment modulates the gut microbiota of the hypercholesterolemic patients. *OMICS* 22 154–163. 10.1089/omi.2017.0130 29432061

[B109] Kim H. Y. Spector A. A. (2018). N-Docosahexaenoylethanolamine: A neurotrophic and neuroprotective metabolite of docosahexaenoic acid. *Mol. Aspects Med.* 64 34–44. 29572109 10.1016/j.mam.2018.03.004

[B110] Kim S. Jazwinski S. M. (2018). The gut microbiota and healthy aging: A mini-review. *Gerontology* 64 513–520. 30025401 10.1159/000490615 PMC6191326

[B111] Kim S. Kim H. Yim Y. S. Ha S. Atarashi K. Tan T. G. (2017). Maternal gut bacteria promote neurodevelopmental abnormalities in mouse offspring. *Nature* 549 528–532. 28902840 10.1038/nature23910 PMC5870873

[B112] Kimura I. Miyamoto J. Ohue-Kitano R. Watanabe K. Yamada T. Onuki M. (2020). Maternal gut microbiota in pregnancy influences offspring metabolic phenotype in mice. *Science* 367:eaaw8429. 10.1126/science.aaw8429 32108090

[B113] Kitajka K. Sinclair A. J. Weisinger R. S. Weisinger H. S. Mathai M. Jayasooriya A. P. (2004). Effects of dietary omega-3 polyunsaturated fatty acids on brain gene expression. *Proc. Natl. Acad. Sci. U.S.A.* 101 10931–10936. 15263092 10.1073/pnas.0402342101 PMC503722

[B114] Knight R. Vrbanac A. Taylor B. C. Aksenov A. Callewaert C. Debelius J. (2018). Best practices for analysing microbiomes. *Nat. Rev. Microbiol.* 16 410–422. 10.1038/s41579-018-0029-9 29795328

[B115] Krol A. Feng G. (2018). Windows of opportunity: Timing in neurodevelopmental disorders. *Curr. Opin. Neurobiol.* 48 59–63. 29125977 10.1016/j.conb.2017.10.014

[B116] Lagadinou M. Onisor M. O. Rigas A. Musetescu D. V. Gkentzi D. Assimakopoulos S. F. (2020). Antimicrobial properties on non-antibiotic drugs in the era of increased bacterial resistance. *Antibiotics (Basel)* 9:107. 10.3390/antibiotics9030107 PMC7175110 32131427

[B117] Ledoux J. (2007). The amygdala. *Curr. Biol.* 17 R868–R874. 17956742 10.1016/j.cub.2007.08.005

[B118] Lense M. D. Ladanyi E. Rabinowitch T. C. Trainor L. Gordon R. (2021). Rhythm and timing as vulnerabilities in neurodevelopmental disorders. *Philos. Trans. R. Soc. Lond. B Biol. Sci.* 376:20200327. 10.1098/rstb.2020.0327 PMC8380970 34420385

[B119] Li M. Wang M. Donovan S. M. (2014). Early development of the gut microbiome and immune-mediated childhood disorders. *Semin. Reprod. Med.* 32 74–86. 24390924 10.1055/s-0033-1361825

[B120] Lin Z. Sur S. Liu P. Li Y. Jiang D. Hou X. (2021). Blood-brain barrier breakdown in relationship to Alzheimer and vascular disease. *Ann. Neurol.* 90 227–238. 34041783 10.1002/ana.26134 PMC8805295

[B121] Liu L. Huh J. R. Shah K. (2022). Microbiota and the gut-brain-axis: Implications for new therapeutic design in the CNS. *EBioMedicine* 77:103908. 10.1016/j.ebiom.2022.103908 PMC8897630 35255456

[B122] Loh J. S. Mak W. Q. Tan L. K. S. Ng C. X. Chan H. H. Yeow S. H. (2024). Microbiota–gut–brain axis and its therapeutic applications in neurodegenerative diseases. *Signal Trans. Targeted Ther.* 9:37. 10.1038/s41392-024-01743-1 PMC10869798 38360862

[B123] Lu J. Synowiec S. Lu L. Yu Y. Bretherick T. Takada S. (2018). Microbiota influence the development of the brain and behaviors in C57BL/6J mice. *PLoS One* 13:e0201829. 10.1371/journal.pone.0201829 PMC6075787 30075011

[B124] Luczynski P. Mcvey Neufeld K. A. Oriach C. S. Clarke G. Dinan T. G. Cryan J. F. (2016a). Growing up in a bubble: Using germ-free animals to assess the influence of the gut microbiota on brain and behavior. *Int. J. Neuropsychopharmacol.* 19:pyw020. 10.1093/ijnp/pyw020 26912607 PMC5006193

[B125] Luczynski P. Whelan S. O. O’sullivan C. Clarke G. Shanahan F. Dinan T. G. (2016b). Adult microbiota-deficient mice have distinct dendritic morphological changes: Differential effects in the amygdala and hippocampus. *Eur. J. Neurosci.* 44 2654–2666. 10.1111/ejn.13291 27256072 PMC5113767

[B126] Machate D. J. Figueiredo P. S. Marcelino G. Guimaraes R. C. A. Hiane P. A. Bogo D. (2020). Fatty acid diets: Regulation of gut microbiota composition and obesity and its related metabolic dysbiosis. *Int. J. Mol. Sci.* 21:4093. 10.3390/ijms21114093 PMC7312778 32521778

[B127] Mallick R. Basak S. Duttaroy A. K. (2019). Docosahexaenoic acid,22:6n-3: Its roles in the structure and function of the brain. *Int. J. Dev. Neurosci.* 79 21–31. 31629800 10.1016/j.ijdevneu.2019.10.004

[B128] Mark K. S. Miller D. W. (1999). Increased permeability of primary cultured brain microvessel endothelial cell monolayers following TNF-alpha exposure. *Life Sci.* 64 1941–1953. 10353592 10.1016/s0024-3205(99)00139-3

[B129] Maseda D. Ricciotti E. (2020). NSAID-gut microbiota interactions. *Front. Pharmacol.* 11:1153. 10.3389/fphar.2020.01153 32848762 PMC7426480

[B130] Maseda D. Zackular J. P. Trindade B. Kirk L. Roxas J. L. Rogers L. M. (2019). Nonsteroidal Anti-inflammatory drugs alter the microbiota and exacerbate Clostridium difficile colitis while dysregulating the inflammatory response. *mBio* 10:e02282-18. 10.1128/mBio.02282-18 30622186 PMC6325247

[B131] Mayassi T. Ladell K. Gudjonson H. Mclaren J. E. Shaw D. G. Tran M. T. (2019). Chronic inflammation permanently reshapes tissue-resident immunity in celiac disease. *Cell* 176 967–981 e919. 10.1016/j.cell.2018.12.039 30739797 PMC6667191

[B132] Menni C. Zierer J. Pallister T. Jackson M. A. Long T. Mohney R. P. (2017). Omega-3 fatty acids correlate with gut microbiome diversity and production of N-carbamylglutamate in middle aged and elderly women. *Sci. Rep.* 7:11079. 10.1038/s41598-017-10382-2 28894110 PMC5593975

[B133] Mittal R. Debs L. H. Patel A. P. Nguyen D. Patel K. O’Connor G. (2017). Neurotransmitters: The critical modulators regulating gut-brain axis. *J. Cell. Physiol.* 232 2359–2372. 10.1002/jcp.25518 27512962 PMC5772764

[B134] Mohammadi A. A. Jazayeri S. Khosravi-Darani K. Solati Z. Mohammadpour N. Asemi Z. (2016). The effects of probiotics on mental health and hypothalamic-pituitary-adrenal axis: A randomized, double-blind, placebo-controlled trial in petrochemical workers. *Nutr. Neurosci.* 19 387–395. 25879690 10.1179/1476830515Y.0000000023

[B135] Moludi J. Khedmatgozar H. Nachvak S. M. Abdollahzad H. Moradinazar M. Sadeghpour Tabaei A. (2022). The effects of co-administration of probiotics and prebiotics on chronic inflammation, and depression symptoms in patients with coronary artery diseases: A randomized clinical trial. *Nutr. Neurosci.* 25 1659–1668. 10.1080/1028415X.2021.1889451 33641656

[B136] Montagne A. Huuskonen M. T. Rajagopal G. Sweeney M. D. Nation D. A. Sepehrband F. (2019). Undetectable gadolinium brain retention in individuals with an age-dependent blood-brain barrier breakdown in the hippocampus and mild cognitive impairment. *Alzheimers Dement.* 15 1568–1575. 10.1016/j.jalz.2019.07.012 31862169 PMC6927478

[B137] Mou Y. Du Y. Zhou L. Yue J. Hu X. Liu Y. (2022). Gut microbiota interact with the brain through systemic chronic inflammation: Implications on neuroinflammation, neurodegeneration, and aging. *Front. Immunol.* 13 796288. 10.3389/fimmu.2022.796288 35464431 PMC9021448

[B138] Mu C. Yang Y. Zhu W. (2016). Gut microbiota: The brain peacekeeper. *Front. Microbiol.* 7:345. 10.3389/fmicb.2016.00345 27014255 PMC4794499

[B139] Mueller N. T. Differding M. K. Zhang M. Maruthur N. M. Juraschek S. P. Miller E. R. III (2021). Metformin affects gut microbiome composition and function and circulating short-chain fatty acids: A randomized trial. *Diab. Care* 44 1462–1471. 10.2337/dc20-2257 34006565 PMC8323185

[B140] Muhammad F. Fan B. Wang R. Ren J. Jia S. Wang L. (2022). The molecular gut-brain axis in early brain development. *Int. J. Mol. Sci.* 23:15389. 10.3390/ijms232315389 PMC9739658 36499716

[B141] Myles I. A. Fontecilla N. M. Janelsins B. M. Vithayathil P. J. Segre J. A. Datta S. K. (2013). Parental dietary fat intake alters offspring microbiome and immunity. *J. Immunol.* 191 3200–3209. 23935191 10.4049/jimmunol.1301057 PMC3831371

[B142] Nagyoszi P. Wilhelm I. Farkas A. E. Fazakas C. Dung N. T. Hasko J. (2010). Expression and regulation of toll-like receptors in cerebral endothelial cells. *Neurochem. Int.* 57 556–564. 20637248 10.1016/j.neuint.2010.07.002

[B143] Naito Y. Kashiwagi K. Takagi T. Andoh A. Inoue R. (2018). Intestinal dysbiosis secondary to proton-pump inhibitor use. *Digestion* 97 195–204. 29316555 10.1159/000481813

[B144] Naseribafrouei A. Hestad K. Avershina E. Sekelja M. Linlokken A. Wilson R. (2014). Correlation between the human fecal microbiota and depression. *Neurogastroenterol. Motil.* 26 1155–1162. 24888394 10.1111/nmo.12378

[B145] Neufeld K. M. Kang N. Bienenstock J. Foster J. A. (2011). Reduced anxiety-like behavior and central neurochemical change in germ-free mice. *Neurogastroenterol. Motil.* 23 255–264 e119. 10.1111/j.1365-2982.2010.01620.x 21054680

[B146] Neuman H. Forsythe P. Uzan A. Avni O. Koren O. (2018). Antibiotics in early life: Dysbiosis and the damage done. *FEMS Microbiol. Rev.* 42 489–499. 29945240 10.1093/femsre/fuy018

[B147] Ogbonnaya E. S. Clarke G. Shanahan F. Dinan T. G. Cryan J. F. O’leary O. F. (2015). Adult hippocampal neurogenesis is regulated by the microbiome. *Biol. Psychiatry* 78 e7–e9. 25700599 10.1016/j.biopsych.2014.12.023

[B148] Ohman L. Dahlen R. Isaksson S. Sjoling A. Wick M. J. Sjovall H. (2013). Serum IL-17A in newly diagnosed treatment-naive patients with ulcerative colitis reflects clinical disease severity and predicts the course of disease. *Inflamm. Bowel Dis.* 19 2433–2439. 10.1097/MIB.0b013e3182a563cb 23966065

[B149] O’Keefe S. J. D. (2016). Diet, microorganisms and their metabolites, and colon cancer. *Nat. Rev. Gastroenterol. Hepatol.* 13 691–706. 10.1038/nrgastro.2016.165 27848961 PMC6312102

[B150] O’mahony S. M. Felice V. D. Nally K. Savignac H. M. Claesson M. J. Scully P. (2014). Disturbance of the gut microbiota in early-life selectively affects visceral pain in adulthood without impacting cognitive or anxiety-related behaviors in male rats. *Neuroscience* 277 885–901. 25088912 10.1016/j.neuroscience.2014.07.054

[B151] O’riordan K. J. Collins M. K. Moloney G. M. Knox E. G. Aburto M. R. Fülling C. (2022). Short chain fatty acids: Microbial metabolites for gut-brain axis signalling. *Mol. Cell Endocrinol.* 546:111572. 10.1016/j.mce.2022.111572 35066114

[B152] Park J. Kim C. H. (2021). Regulation of common neurological disorders by gut microbial metabolites. *Exp. Mol. Med.* 53 1821–1833. 34857900 10.1038/s12276-021-00703-x PMC8741890

[B153] Pauwels I. Versporten A. Drapier N. Vlieghe E. Goossens H. Global P. P. S. N. (2021). Hospital antibiotic prescribing patterns in adult patients according to the WHO Access, Watch and Reserve classification (AWaRe): Results from a worldwide point prevalence survey in 69 countries. *J. Antimicrob. Chemother.* 76 1614–1624. 10.1093/jac/dkab050 33822971 PMC8120336

[B154] Pirbaglou M. Katz J. de Souza R. J. Stearns J. C. Motamed M. Ritvo P. (2016). Probiotic supplementation can positively affect anxiety and depressive symptoms: A systematic review of randomized controlled trials. *Nutr. Res.* 36 889–898. 10.1016/j.nutres.2016.06.009 27632908

[B155] Rao M. Gershon M. D. (2018). Enteric nervous system development: What could possibly go wrong? *Nat. Rev. Neurosci.* 19 552–565. 10.1038/s41583-018-0041-0 30046054 PMC6261281

[B156] Raygan F. Ostadmohammadi V. Bahmani F. Asemi Z. (2018). The effects of vitamin D and probiotic co-supplementation on mental health parameters and metabolic status in type 2 diabetic patients with coronary heart disease: A randomized, double-blind, placebo-controlled trial. *Prog. Neuropsychopharmacol. Biol. Psychiatry* 84 50–55. 10.1016/j.pnpbp.2018.02.007 29432877

[B157] Rhee S. H. Pothoulakis C. Mayer E. A. (2009). Principles and clinical implications of the brainngut enteric microbiota axis. *Nat. Rev. Gastroenterol. Hepatol.* 6 306–314. 10.1038/nrgastro.2009.35 19404271 PMC3817714

[B158] Rincel M. Darnaudery M. (2020). Maternal separation in rodents: A journey from gut to brain and nutritional perspectives. *Proc. Nutr. Soc.* 79 113–132. 10.1017/S0029665119000958 31250784

[B159] Rinninella E. Raoul P. Cintoni M. Franceschi F. Miggiano G. D. Gasbarrini A. (2019). What is the healthy gut microbiota composition? A changing ecosystem across age, environment, diet, and diseases. *Microorganisms* 7:14. 10.3390/microorganisms7010014 PMC6351938 30634578

[B160] Rogers G. B. Keating D. J. Young R. L. Wong M. L. Licinio J. Wesselingh S. (2016). From gut dysbiosis to altered brain function and mental illness: Mechanisms and pathways. *Mol. Psychiatry* 21 738–748. 27090305 10.1038/mp.2016.50 PMC4879184

[B161] Rogers M. M. Aronoff D. M. (2016). The influence of non-steroidal anti-inflammatory drugs on the gut microbiome. *Clin. Microbiol. Infect.* 22 178 e171–178 e179. 10.1016/j.cmi.2015.10.003 PMC4754147 26482265

[B162] Roubaud-Baudron C. Ruiz V. E. Swan A. M. Jr. Vallance B. A. Ozkul C. Pei Z. (2019). Long-term effects of early-life antibiotic exposure on resistance to subsequent bacterial infection. *mBio* 10 e2820–e2819. 10.1128/mBio.02820-19 PMC6935859 31874917

[B163] Rutsch A. Kantsjo J. B. Ronchi F. (2020). The gut-brain axis: How microbiota and host inflammasome influence brain physiology and pathology. *Front. Immunol.* 11:604179. 10.3389/fimmu.2020.604179 33362788 PMC7758428

[B164] Sampei S. Okada H. Tomita H. Takada C. Suzuki K. Kinoshita T. (2021). Endothelial glycocalyx disorders may be associated with extended inflammation during endotoxemia in a diabetic mouse Model. *Front. Cell Dev. Biol.* 9:623582. 10.3389/fcell.2021.623582 33869173 PMC8047120

[B165] Sampson T. R. Mazmanian S. K. (2015). Control of brain development, function, and behavior by the microbiome. *Cell Host Microbe* 17 565–576. 25974299 10.1016/j.chom.2015.04.011 PMC4442490

[B166] Sampson T. R. Debelius J. W. Thron T. Janssen S. Shastri G. G. Ilhan Z. E. (2016). Gut microbiota regulate motor deficits and neuroinflammation in a model of Parkinson’s disease. *Cell* 167 1469–1480 e1412. 10.1016/j.cell.2016.11.018 27912057 PMC5718049

[B167] Sánchez-Campillo M. Ruiz-Palacios M. Ruiz-Alcaraz A. J. Prieto-Sánchez M. T. Blanco-Carnero J. E. Zornoza M. (2020). Child head circumference and placental MFSD2a expression are associated to the level of MFSD2a in maternal blood during pregnancy. *Front. Endocrinol.* 11 1–11. 10.3389/fendo.2020.00038 32117064 PMC7012934

[B168] Sanchorawala V. Palladini G. Kukreti V. Zonder J. A. Cohen A. D. Seldin D. C. (2017). A phase 1/2 study of the oral proteasome inhibitor ixazomib in relapsed or refractory AL amyloidosis. *Blood* 130 597–605. 28550039 10.1182/blood-2017-03-771220 PMC6911836

[B169] Savignac H. M. Dinan T. G. Cryan J. F. (2011). Resistance to early-life stress in mice: Effects of geneticbackground and stress duration. *Front. Behav. Neurosci.* 5:13. 10.3389/fnbeh.2011.00013 21519375 PMC3075880

[B170] Schumann C. M. Amaral D. G. (2006). Stereological analysis of amygdala neuron number in autism. *J. Neurosci.* 26 7674–7679. 16855095 10.1523/JNEUROSCI.1285-06.2006 PMC6674270

[B171] Senatorov V. V. Jr. Friedman A. R. Milikovsky D. Z. Ofer J. Saar-Ashkenazy R. Charbash A. (2019). Blood-brain barrier dysfunction in aging induces hyperactivation of TGFbeta signaling and chronic yet reversible neural dysfunction. *Sci. Transl. Med.* 11:eaaw8283. 10.1126/scitranslmed.aaw8283 31801886

[B172] Shale M. Schiering C. Powrie F. (2013). CD4(+) T-cell subsets in intestinal inflammation. *Immunol. Rev.* 252 164–182. 23405904 10.1111/imr.12039 PMC3736165

[B173] Sharon G. Sampson T. R. Geschwind D. H. Mazmanian S. K. (2016). The central nervous system and the gut microbiome. *Cell* 167 915–932. 27814521 10.1016/j.cell.2016.10.027 PMC5127403

[B174] Shin Yim Y. Park A. Berrios J. Lafourcade M. Pascual L. M. Soares N. (2017). Reversing behavioural abnormalities in mice exposed to maternal inflammation. *Nature* 549 482–487. 10.1038/nature23909 28902835 PMC5796433

[B175] Silva Y. P. Bernardi A. Frozza R. L. (2020). The role of short-chain fatty acids from gut microbiota in gut-brain communication. *Front. Endocrinol. (Lausanne)* 11:25. 10.3389/fendo.2020.00025 PMC7005631 32082260

[B176] Sirisinha S. (2016). The potential impact of gut microbiota on your health: Current status and future challenges. *Asian Pac. J. Allergy Immunol.* 34 249–264. 10.12932/AP0803 28042926

[B177] Skonieczna Zydecka K. Marlicz W. Misera A. Koulaouzidis A. Aoniewski I. (2018). Microbiome the missing link in the gut-brain axis: Focus on its role in gastrointestinal and mental health. *J. Clin. Med.* 7:521. 10.3390/jcm7120521 30544486 PMC6306769

[B178] Socala K. Doboszewska U. Szopa A. Serefko A. Wlodarczyk M. Zielinska A. (2021). The role of microbiota-gut-brain axis in neuropsychiatric and neurological disorders. *Pharmacol. Res.* 172:105840. 10.1016/j.phrs.2021.105840 34450312

[B179] Sokol H. Pigneur B. Watterlot L. Lakhdari O. Bermudez-Humaran L. G. Gratadoux J. J. (2008). Faecalibacterium prausnitzii is an anti-inflammatory commensal bacterium identified by gut microbiota analysis of Crohn disease patients. *Proc. Natl. Acad. Sci. U.S.A.* 105 16731–16736. 18936492 10.1073/pnas.0804812105 PMC2575488

[B180] Sokol H. Seksik P. Furet J. P. Firmesse O. Nion-Larmurier I. Beaugerie L. (2009). Low counts of Faecalibacterium prausnitzii in colitis microbiota. *Inflamm. Bowel. Dis.* 15 1183–1189. 19235886 10.1002/ibd.20903

[B181] Srinivas V. Varma S. Kona S. R. Ibrahim A. Duttaroy A. K. Basak S. (2023). Dietary omega-3 fatty acid deficiency from pre-pregnancy to lactation affects expression of genes involved in hippocampal neurogenesis of the offspring. *Prostagl. Leukotrienes Essent. Fatty Acids* 191:102566. 10.1016/j.plefa.2023.102566 36924605

[B182] Sudo N. Chida Y. Aiba Y. Sonoda J. Oyama N. Yu X. N. (2004). Postnatal microbial colonization programs the hypothalamic pituitary adrenal system for stress response in mice. *J. Physiol.* 558 263–275. 10.1113/jphysiol.2004.063388 15133062 PMC1664925

[B183] Surendar J. Aravindhan V. Rao M. M. Ganesan A. Mohan V. (2011). Decreased serum interleukin-17 and increased transforming growth factor-beta levels in subjects with metabolic syndrome (Chennai Urban Rural Epidemiology Study-95). *Metabolism* 60 586–590. 10.1016/j.metabol.2010.06.003 20667562

[B184] Svensson E. Horvath-Puho E. Thomsen R. W. Djurhuus J. C. Pedersen L. Borghammer P. (2015). Vagotomy and subsequent risk of Parkinson’s disease. *Ann Neurol* 78 522–529. 26031848 10.1002/ana.24448

[B185] Tang M. Zhang M. Wang L. Li H. Cai H. Dang R. (2018). Maternal dietary of n-3 polyunsaturated fatty acids affects the neurogenesis and neurochemical in female rat at weaning. *Prostagl. Leukot Essent. Fatty Acids* 128 11–20. 10.1016/j.plefa.2017.11.001 29413357

[B186] Taylor A. M. Holscher H. D. (2020). A review of dietary and microbial connections to depression, anxiety, and stress. *Nutr. Neurosci.* 23 237–250. 29985786 10.1080/1028415X.2018.1493808

[B187] Thevaranjan N. Puchta A. Schulz C. Naidoo A. Szamosi J. C. Verschoor C. P. (2017). Age-associated microbial dysbiosis promotes intestinal permeability, systemic inflammation, and macrophage dysfunction. *Cell Host Microbe* 21 455–466.e454. 28407483 10.1016/j.chom.2017.03.002 PMC5392495

[B188] Thielke K. H. Hoffmann-Moujahid A. Weisser C. Waldkirch E. Pabst R. Holtmeier W. (2003). Proliferating intestinal gamma/delta T cells recirculate rapidly and are a major source of the gamma/delta T cell pool in the peripheral blood. *Eur. J. Immunol.* 33 1649–1656. 10.1002/eji.200323442 12778483

[B189] Tochitani S. Ikeno T. Ito T. Sakurai A. Yamauchi T. Matsuzaki H. (2016). Administration of non-absorbable antibiotics to pregnant mice to perturb the maternal gut microbiota is associated with alterations in offspring behavior. *PLoS One* 11:e0138293. 10.1371/journal.pone.0138293 26789865 PMC4720425

[B190] Tong X. Xu J. Lian F. Yu X. Zhao Y. Xu L. (2018). Structural alteration of gut microbiota during the amelioration of human type 2 diabetes with hyperlipidemia by metformin and a traditional chinese herbal formula: A multicenter, randomized, open label clinical trial. *mBio* 9 e2392–e2317. 10.1128/mBio.02392-17 29789365 PMC5964358

[B191] Tremlett H. Bauer K. C. Appel-Cresswell S. Finlay B. B. Waubant E. (2017). The gut microbiome in human neurological disease: A review. *Ann. Neurol.* 81 369–382. 28220542 10.1002/ana.24901

[B192] Turnbaugh P. J. Ley R. E. Mahowald M. A. Magrini V. Mardis E. R. Gordon J. I. (2006). An obesity-associated gut microbiome with increased capacity for energy harvest. *Nature* 444 1027–1031. 17183312 10.1038/nature05414

[B193] Van Ameringen M. Turna J. Patterson B. Pipe A. Mao R. Q. Anglin R. (2019). The gut microbiome in psychiatry: A primer for clinicians. *Depress Anxiety* 36 1004–1025. 31356715 10.1002/da.22936

[B194] Van Den Elsen L. W. J. Verhasselt V. (2021). Human milk drives the intimate interplay between gut immunity and adipose tissue for healthy growth. *Front. Immunol.* 12:645415. 10.3389/fimmu.2021.645415 33912171 PMC8071867

[B195] Venkataraman R. Madempudi R. S. Neelamraju J. Ahire J. J. Vinay H. R. Lal A. (2021). Effect of multi-strain probiotic formulation on students facing examination stress: A double-blind, placebo-controlled study. *Probiotics Antimicrob. Proteins* 13 12–18. 10.1007/s12602-020-09681-4 32601955

[B196] Vernocchi P. Del Chierico F. Putignani L. (2016). Gut microbiota profiling: Metabolomics based approach to unravel compounds affecting human health. *Front. Microbiol.* 7:1144. 10.3389/fmicb.2016.01144 PMC4960240 27507964

[B197] Vieira-Silva S. Falony G. Belda E. Nielsen T. Aron-Wisnewsky J. Chakaroun R. (2020). Statin therapy is associated with lower prevalence of gut microbiota dysbiosis. *Nature* 581 310–315. 10.1038/s41586-020-2269-x 32433607

[B198] Vuong H. E. Pronovost G. N. Williams D. W. Coley E. J. L. Siegler E. L. Qiu A. (2020). The maternal microbiome modulates fetal neurodevelopment in mice. *Nature* 586 281–286. 32968276 10.1038/s41586-020-2745-3 PMC7554197

[B199] Vuong H. E. Yano J. M. Fung T. C. Hsiao E. Y. (2017). The microbiome and host behavior. *Annu. Rev. Neurosci.* 40 21–49. 28301775 10.1146/annurev-neuro-072116-031347 PMC6661159

[B200] Waisman A. Hauptmann J. Regen T. (2015). The role of IL-17 in CNS diseases. *Acta Neuropathol.* 129 625–637. 25716179 10.1007/s00401-015-1402-7

[B201] Wan X. Eguchi A. Sakamoto A. Fujita Y. Yang Y. Qu Y. (2023). Impact of broad-spectrum antibiotics on the gut-microbiota-spleen-brain axis. *Brain Behav. Immun. Health* 27:100573. 10.1016/j.bbih.2022.100573 36583066 PMC9793168

[B202] Warren A. Nyavor Y. Zarabian N. Mahoney A. Frame L. A. (2024). The microbiota-gut-brain-immune interface in the pathogenesis of neuroinflammatory diseases: A narrative review of the emerging literature. *Front. Immunol.* 15:1365673. 10.3389/fimmu.2024.1365673 38817603 PMC11137262

[B203] Wauters L. Tito R. Y. Ceulemans M. Lambaerts M. Accarie A. Rymenans L. (2021). Duodenal dysbiosis and relation to the efficacy of proton pump inhibitors in functional dyspepsia. *Int. J. Mol. Sci.* 22:13609. 10.3390/ijms222413609 34948413 PMC8708077

[B204] Wood J. D. Galligan J. J. (2004). Function of opioids in the enteric nervous system. *Neurogastroenterol. Motilit.* 16 17–28. 10.1111/j.1743-3150.2004.00554.x 15357848

[B205] Wu H. Esteve E. Tremaroli V. Khan M. T. Caesar R. Manneras-Holm L. (2017). Metformin alters the gut microbiome of individuals with treatment-naive type 2 diabetes, contributing to the therapeutic effects of the drug. *Nat. Med.* 23 850–858. 10.1038/nm.4345 28530702

[B206] Xu H. Li H. (2019). Acne, the skin microbiome, and antibiotic treatment. *Am. J. Clin. Dermatol.* 20 335–344. 30632097 10.1007/s40257-018-00417-3 PMC6534434

[B207] Yacyshyn B. Meddings J. Sadowski D. Bowen-Yacyshyn M. B. (1996). Multiple sclerosis patients have peripheral blood CD45RO+ B cells and increased intestinal permeability. *Dig. Dis. Sci.* 41 2493–2498. 10.1007/BF02100148 9011463

[B208] Yang C. Qiao Z. Xu Z. Wang X. Deng Q. Chen W. (2021). Algal Oil rich in docosahexaenoic acid alleviates intestinal inflammation induced by antibiotics associated with the modulation of the gut microbiome and metabolome. *J. Agric. Food Chem.* 69 9124–9136. 10.1021/acs.jafc.0c07323 33900083

[B209] Yano J. M. Yu K. Donaldson G. P. Shastri G. G. Ann P. Ma L. (2015). Indigenous bacteria from the gut microbiota regulate host serotonin biosynthesis. *Cells* 161 264–276. 10.1016/j.cell.2015.02.047 25860609 PMC4393509

[B210] Zadori Z. S. Kiraly K. Al-Khrasani M. Gyires K. (2023). Interactions between NSAIDs, opioids and the gut microbiota - future perspectives in the management of inflammation and pain. *Pharmacol. Ther.* 241:108327. 10.1016/j.pharmthera.2022.108327 36473615

[B211] Zhang H. Chen Y. Wang Z. Xie G. Liu M. Yuan B. (2022). Implications of gut microbiota in neurodegenerative diseases. *Front. Immunol.* 13:785644. 10.3389/fimmu.2022.785644 PMC8882587 35237258

